# The TRACERS Fluxgate Magnetometer (MAG)

**DOI:** 10.1007/s11214-025-01212-3

**Published:** 2025-09-12

**Authors:** Robert J. Strangeway, Hao Cao, Eric Orrill, Ryan P. Caron, David Pierce, Ryan Seaton, Henry H. Gonzalez, Enrique Gurrola, William Greer, David Leneman, Michael J. Lawson, Vicente Capistrano, Dean Bushong, Jianxin Chen, Cynthia L. Russell, Jiashu Wu, David M. Miles, Craig A. Kletzing

**Affiliations:** 1 Department of Earth, Planetary, and Space Sciences, University of California, Los Angeles, Los Angeles, CA USA; 2 Timoneer Aerospace LLC, Pheonix, AZ USA; 3 AVARA Magnetics LLC, Las Vegas, NV USA; 4 Blue Origin, Kent, WA USA; 5 Orbiting Engineers, Gilbert, AZ USA; 6 Baja Technology LLC, Tempe, AZ USA; 7 Department of Physics and Astronomy, University of Iowa, Iowa City, IA USA

**Keywords:** Heliophysics, Magnetosphere-ionosphere, Magnetic fields, Fluxgate

## Abstract

The NASA Tandem Reconnection and Cusp Electrodynamics Reconnaissance Satellites (TRACERS) mission is a two-spacecraft mission designed to explore the temporal and spatial signatures of magnetic reconnection as observed at the low altitude dayside cusp. The instrumentation on each TRACERS spacecraft includes a three-axis vector fluxgate magnetometer (MAG). The MAG sensor design heritage is from Magnetospheric Multiscale (MMS), while the electronics heritage is from the InSight mission to Mars. Testing as part of the MAG instrument delivery verified that the MAG dynamic range exceeded ±60,000 nT with a resolution of ∼9 pT to provide margin. The fluxgate magnetometers have been calibrated on the ground, but as is typical for fluxgates they will be re-calibrated using on-orbit data. The TRACERS spacecraft are spinning spacecraft in an orbit at 590 km altitude. Absolute gains, orientation, and spin-axis offsets will be determined through comparison with the International Geomagnetic Reference Field (IGRF) with an underlying orbit-period cadence. Additionally, spin-tones allow determination of relative angular orientation and gain and spin-plane offsets at spin-period temporal resolution. To meet the TRACERS mission science objectives MAG will measure magnetic field perturbations from large scale field-aligned currents, and shorter scale Alfvén waves. The electromagnetic energy flux associated with these magnetic field perturbations has major impacts on particle acceleration along the flux tube and ionospheric heating through Joule dissipation. This conversion from electromagnetic to particle energy is a primary driver for the escape of ionospheric plasma into the magnetosphere, making this an important secondary science objective for the TRACERS mission.

## Introduction

The Tandem Reconnection and Cusp Electrodynamics Reconnaissance Satellites (TRACERS) mission is a two-spacecraft mission designed to resolve the temporal versus spatial variability of magnetic reconnection as observed at low altitude (590 km) in the dayside cusp region (Miles et al. 2025a). As discussed by Miles et al., the TRACERS mission has three primary science objectives. Science Objective 1 (SO1) is to “[d]etermine whether magnetopause reconnection is primarily spatially or temporally variable for a range of solar wind conditions.” Science Objective 2 (SO2) follows on from SO1, “[f]or temporally varying reconnection, determine how the reconnection rate evolves.” Lastly, Science Objective 3 (SO3) is to “[d]etermine to what extent dynamic structures in the cusp are associated with temporal versus spatial reconnection.” SO1 and SO2 are primarily addressed through the ion and electron particle detectors, and measurements of the plasma convection speed as determined by the electric field instrument measurements. Low frequency magnetic field measurements are support for SO1 and SO2, but are not required for closure. For SO3 the fluxgate magnetometer provides the magnetospheric context for the other instrument measurements, especially by providing the detailed structure of the low latitude cusp region through measurements of Field-Aligned Currents (FACs). Consequently, SO3 is the main driver for the magnetometer instrument requirements, as discussed in the next section.

One other aspect of the TRACERS mission that leads to fluxgate magnetometer instrument requirements is the separation of each TRACERS orbit into a Region of Interest (ROI) and the Back Orbit (BOR). The ROI is nominally centered around the expected location of the cusp, as described by Petrinec et al. (2025). For the ROI all the instruments are operating, and the fluxgate magnetometer (MAG) acquires data at 128 samples per second (sps), giving a high enough frequency rate to allow for sampling small-scale (∼1 km) spatial structures. In the BOR the instruments operate at a lower cadence. For MAG, since part of the on-orbit calibration process (see Sect. 5) requires trending against the Earth’s magnetic field, a full orbit of data is desirable. The MAG BOR sampling rate is chosen to be 16 sps.

The order of this paper is as follows. The next section discusses the science objectives and the resultant instrument requirements for the fluxgate magnetometer (MAG). Section 3 outlines the heritage and design of the TRACERS MAG. The section also includes some photographs of flight hardware. Following this, Sect. 4 discusses the derived instrument requirements as flown down from the TRACERS project, and how they were verified. The data used to verify the requirements are also the basis for the pre-launch calibration of the instrument. As noted in Sect. 4, the pre-launch calibration is considered preliminary, and Sect. 5 gives the approach to post-launch on-orbit calibration. This leads into Sect. 6 where we describe post-launch operations and data production. Section 7 is a discussion of additional science opportunities enabled by the TRACERS mission, and Sect. 8 presents our concluding remarks.

## Science Objectives and Instrument Requirements for the Magnetic Field Investigation

### Magnetic Field Perturbations and TRACERS Science

As noted in the introduction, the primary science objective of the TRACERS MAG is to provide high temporal and spatial resolution measurements of the magnetic fields associated with Field-Aligned Currents (FACs). FACs can generally be separated into two types: large-scale and small-scale. An example of the former are the Region-1 and Region-2 current systems (Iijima and Potemra 1976a) that are most clearly resolved away from noon and midnight local times. Near midnight the currents overlap, around the Harang discontinuity (Heppner 1972), while Iijima and Potemra (1976b) suggested the presence of an additional current system near noon (see their Fig. 6). Above the ionosphere the magnetic field of these large-scale current systems can be related to the corresponding electric field through the height integrated Pedersen conductivity by the well-known equation $\delta B_{\bot} = \mu _{0} \Sigma _{p} E_{\bot} $, where $\delta B_{\bot} $ is the magnetic field perturbation associated with the current system, $E_{\bot} $ is the perpendicular electric field, and $\Sigma _{p}$ is the height integrated Pedersen conductivity.

**Fig. 1:**
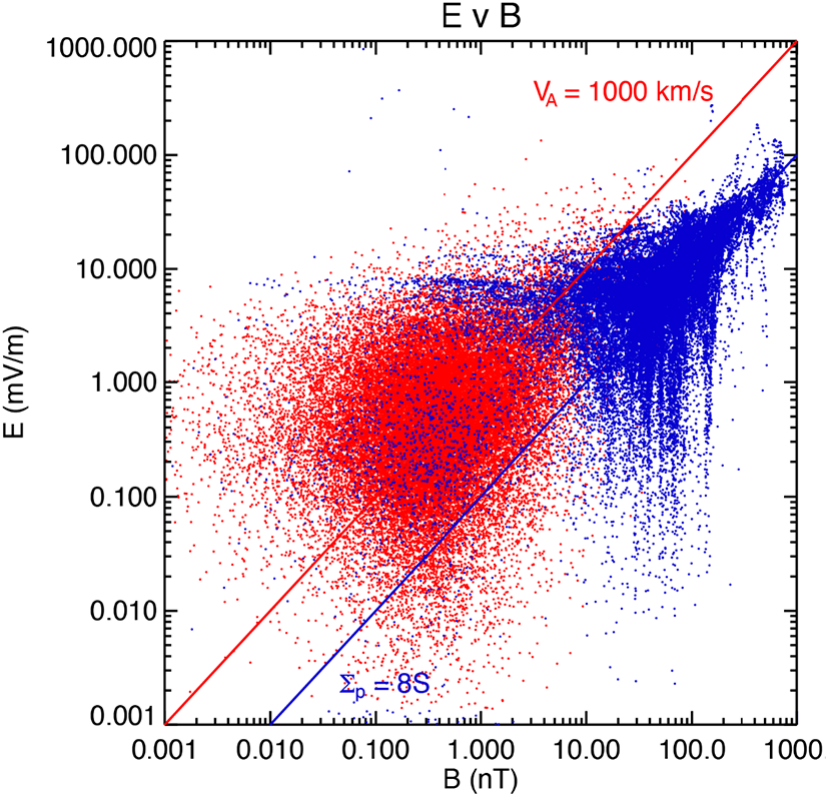
Electric and magnetic field perturbations as observed at 4000 km by the FAST spacecraft separated into low-pass (< ∼0.1 Hz) and high-pass (> ∼0.1 Hz and < 0.5 Hz) (after Strangeway et al. 2005)

By way of contrast, for sufficiently high frequencies (and, by inference, for sufficiently small spatial scales), the electric and magnetic field are related through the local Alfvén speed ($V_{A}$): $E_{\bot} = V_{A} \delta B_{\bot} $. Indeed, the ratio of the two effective height-integrated conductivities $R= \mu _{0} \Sigma _{p} V_{A}$ is an important parameter in understanding wave propagation and transmission in the topside ionosphere (see, for example, Lysak (1999) and references therein). In general $R\neq 1$, and this means that an incident Alfvén wave will be reflected. But this also means that, for an Alfvén wave of sufficiently long wavelength (i.e., low frequency), the $E_{\bot} / \delta B_{\bot} $ ratio above the ionosphere is determined by ${1} / {\mu _{0} \Sigma _{p}}$, not $V_{A}$. That is, the wave measurements are in the “near field” of the ionospherically reflected wave, where the wave fields are the sum of both the incident and reflected waves. At altitudes sufficiently far from the ionosphere the wave fields tend to separate from incident to reflected, mainly because of the plasma motion. The wave propagation is within the frame of the convecting plasma. Evidence of this separation can be seen in Wygant et al. (2002), for example, who argue that at times the wave-related Poynting flux is sufficient to power the aurora. For FAST, the transition from ${1} / {\mu _{0} \Sigma _{p}}$ to $V_{A}$ for $E_{\bot} / \delta B_{\bot} $ was found to occur around 0.1 Hz as shown in Fig. 1, after Strangeway et al. (2005). As discussed in Appendix A2 of Strangeway et al. (2005), FAST magnetometer data came at a variety of rates, depending on instrument commanding. For Fig. 1 the data were averaged down to 1 sps using running box-car averages. A 7-point running box-car average was then applied to these 1 sps data to provide a “DC” measurement. The difference between the 1 sps data and the DC data were used to specify the amplitude of the low frequency waves, with a 0.5 Hz Nyquist frequency and an approximate low frequency cut-off at 0.1 Hz. The low frequency cut-off at 0.1 Hz marks where the power of the waves generally exceeds the DC power.

In Fig. 1 DC signals are indicated by blue, while the ∼0.1 Hz to 0.5 Hz signals are indicated by red. The diagonal lines show that the high-pass signals are consistent with an Alfvén speed of the order 1000 km/s, i.e., wavelengths < 10000 km for frequencies > 0.1 Hz. FAST was at 4000 km altitude. This implies that at TRACERS altitudes (∼ 400 km above the E- and lower F-region) the transition would be about a factor ten higher in frequency, around 1 Hz. (This does not take into account any Doppler shift due to spacecraft motion through short perpendicular wavelength structures, although this is less relevant for electromagnetic waves that tend to have longer wavelengths.)

### Derived MAG Instrument Requirements

Figure 1 also provides a useful basis for indicating how the MAG performance requirements were determined. The emphasis here is on what is required for MAG to meet Science Objective 3 (SO3). These are listed Table 1. In the Table we also indicate the actual instrument performance, rather than repeat the table later in the paper. How these requirements were verified is described in more detail in Sect. 4.

**Table 1:** Fluxgate magnetometer requirements for SO3 and actual performance during ground tests

Parameter	Requirement	Actual Performance
Dynamic Range	± 60,000 nT	Better than ± 68,000 nT
Resolution	0.25 nT	Better than 0.01 nT
Time Resolution	> 10 sps	128 sps (ROI) 16 sps (BOR)
Alignment Knowledge	< 1°	< 0.5°
Sensitivity (noise level)	100 pT/$\sqrt{\phantom{|}}$Hz at 1 Hz	100 pT/$\sqrt{\phantom{|}}$Hz at 1 Hz
Accuracy	Better than 100 nT(25 nT offset per axis for MAG^∗^)	< 22 nT per axis

^∗^See discussion below

The first requirement is the dynamic range of the instrument. Since TRACERS is in a low ∼600 km altitude orbit, the ambient magnetic field is expected to be around 50,000 nT in magnitude, the requirement of ± 60,000 nT allows for some margin.

In specifying the resolution requirement Fig. 1 shows that the majority of the signals at FAST altitudes are greater than 0.1 nT. Given the difference in altitude between FAST and TRACERS, the flux-tube area is about a factor 5 smaller at TRACERS, and the mapped lower limit would be of order 0.2 nT. Similarly, the frequency range of the FAST data shown in Fig. 1 was sufficient to resolve large-scale signals versus Alfvénic waves. Because the altitude of TRACERS wrt the E- and lower F-region ionosphere is ∼ factor of 10 smaller than FAST (∼400 km v. ∼4000 km), the frequency for the transition increases by a factor 10. That is 10 sps for a 5 Hz Nyquist frequency. As discussed in the next section, based on the Magnetospheric Multiscale (MMS) flight heritage, the UCLA fluxgate magnetometer is designed to provide data at 128 sps, and this rate is used for the ROI, with an 8-point box-car average used to provide the BOR data at 16 sps.

The alignment knowledge requirement is mainly driven by the need to align the MAG measurements with the other fields instruments on TRACERS (The Magnetic Search Coil, MSC, and the Electric Field Instrument, EFI). The alignment angles specify the orientation of the sensor sense-axes relative to the magnetometer coordinate system, which is defined in the spacecraft frame.

We consider the noise requirement and sensitivity to be equivalent, since any naturally occurring signal becomes difficult to measure if the Power Spectral Density (PSD) of the signal falls below the instrument noise level. The noise requirement is partially justified by the science objectives and depends to some extent on the signal being measured. Highly coherent signals always have better signal to noise ratios than more turbulent signals. Furthermore, the TRACERS MAG will fly in a low altitude orbit, where the dynamic range is large. As such, the dynamic range tends to drive the instrument noise, rather than any intrinsic property of the ring cores. It is worth noting, however, that at 1 Hz the PSD, which is given by the square of the amplitude, is 0.01 nT^2^/Hz. Below a few Hz the PSD of the instrument noise varies as 1/f. Integrating the PSD from a period of 0.1 seconds (10 Hz) to 1000 seconds (0.001 Hz), with the latter encompassing the duration of the spacecraft passage through the ROI, then the RMS amplitude of the corresponding signal is 0.30 nT, comparable to the required resolution.

In standard engineering parlance accuracy is usually taken to specify how well the average of a series of measurements represents the actual value of the underlying observable, while precision reflects the standard deviation of the average. Within the context of the magnetic field measurements on TRACERS the accuracy is best described by the offsets in the magnetic field measurements, while precision is more related to the gain.

As noted in the introduction, in the context of MAG, TRACERS SO3 centers on the signatures of Field-Aligned Currents (FACs), be they large scale (quasi-static), or Alfvénic. Again, Fig. 1 shows magnetic fields as observed by the FAST spacecraft and large scale FACs extend from ∼ 1 nT to several 100 s of nT, while the Alfvénic magnetic fields extend from ∼ 0.01 nT to ∼ 10 nT. At first sight, then, the accuracy requirement would be 1 nT or even lower. But this not a feasible requirement for a class D mission such as TRACERS. In addition, since TRACERS is a spinner, spin-plane offsets can be determined at a high (few spin-period) cadence (see Elphic et al. 2001 and Sect. 5 for examples from FAST). The accuracy requirement should consequently be considered to be a requirement on the magnitude of the allowed net offset. Early in the project, during Phase A, it was decided that 100 nT would be a reasonable accuracy requirement and became the baseline for the TRACERS mission level magnetometer requirements. TRACERS also implemented a Magnetics Control Board (MCB) to manage a magnetic cleanliness program (see Miles et al. 2025b). The magnetic cleanliness program identified materials that should be avoided from a magnetics perspective, assessed system designs (e.g., solar cell geometry in the solar arrays), and established a screening program.

It should be noted that an accuracy requirement had to be specified and the 100 nT net offset was chosen as a compromise. A smaller value (say 10 nT) would be very hard to achieve, as noted in Table 1 the magnetometer offsets alone exceed this value. On the other hand, a larger requirement (1000 nT) would be comparable to the largest field-aligned current signatures, and the on-orbit offset determination would be less reliable, compromising the ability to measure Alfvén-wave signals.

100 nT is the net offset magnitude and includes the contributions from MAG itself and the other systems and components on the spacecraft. For MAG 25 nT per axis was chosen, which is consistent with our experience with FAST. This corresponds to an offset magnitude of 43 nT, allowing a 90 nT net offset for the remainder, using a root sum squared approach to combining the two, $\sqrt{\phantom{|}}$(43^2^ + 90^2^) = 100 to the nearest integer.

## Heritage and Design of the TRACERS MAG Instrument

### Heritage

**Fig. 2:**
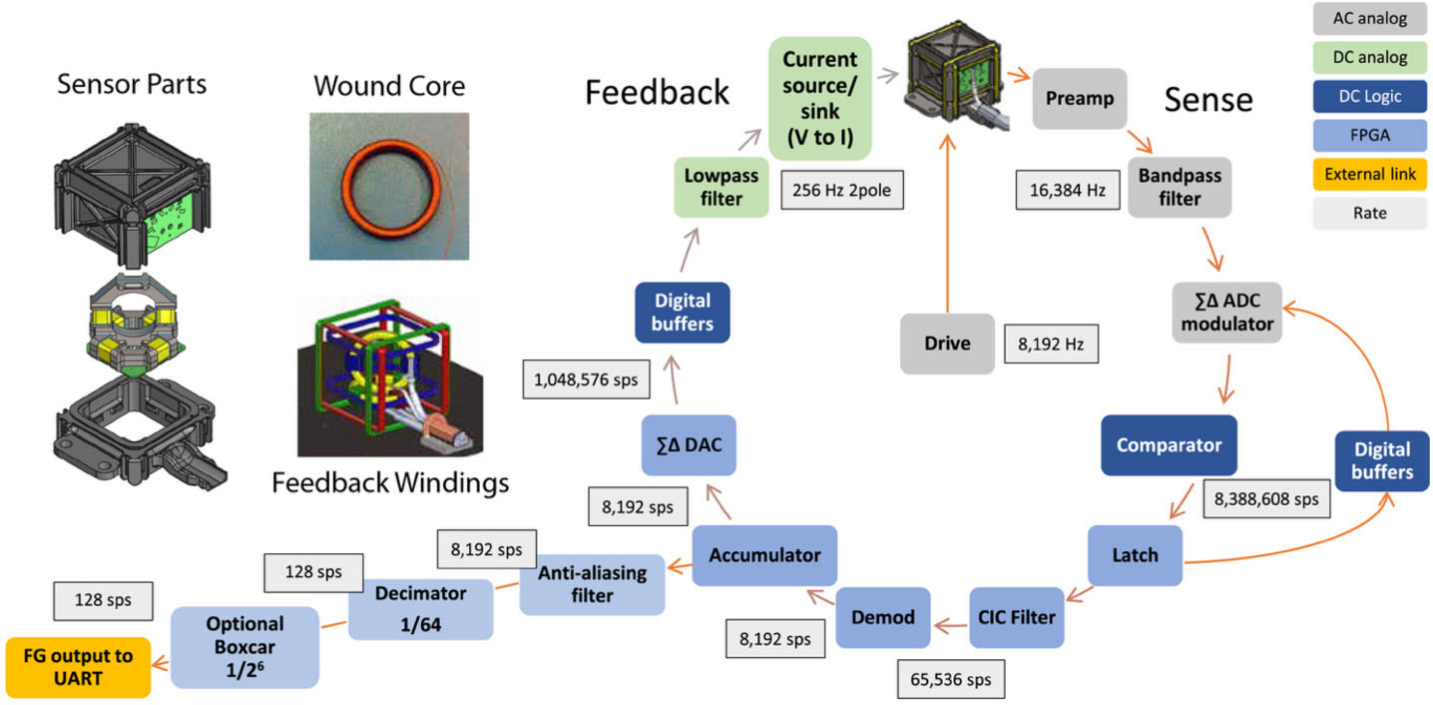
Fluxgate sensor parts (upper left) and the electronics block diagram (right hand side and lower part)

As an institution UCLA has a long history of building flight-qualified magnetometers, going back to the late 1960s with the ATS 1 and OGO 5 missions. In terms of scientific heritage, UCLA has familiarity with low altitude high inclination spacecraft, notably FAST (Elphic et al. 2001), and Space Technology 5 (ST5, Slavin et al. 2008), both of which had apogee near 4000 km altitude. Indeed, the lessons learned from FAST on-orbit calibration will be applied to the TRACERS mission. One of the objectives for ST5, as a technology demonstration project, was to develop a low mass fluxgate sensor design by using only two ring cores, instead of the standard three ring core design (one ring core per axis). The two ring core design was the basis for the MMS sensors, but for MMS the two ring cores were high quality 1” diameter ring cores, rather than the lower quality 5/8” and 3/8” diameter ring cores used for ST5. The sensor armature and copper windings were also redesigned to be less massive and use Polyether Ether Ketone (PEEK) plastic as the material (see Russell et al. 2016). A schematic showing the sensor design is presented in the upper left of Fig. 2.

The heritage for the electronics follows a slightly different path. We include a detailed block diagram for UCLA’s fluxgate magnetometer to place that heritage in context. Before going into details, at the top-level a fluxgate magnetometer works by first driving a ring core into and out of saturation (the grey box labelled “Drive” in the middle of the circular part of the flow chart), using the copper windings around the ring core (on the left). The yellow taped windings in the “Sensor Parts” model are used to sense the changing magnetic induction. The right half of the block diagram shows the “Sense” circuit function. The sense signal is then processed via a Field Programmable Gate Array (FPGA) that provides data to the Central Data Processing Unit (CDPU), via the path at lower left, and forms part of the path that generates a “Feedback” signal that cancels the external signal, via the left-hand side of the circular path. By using feedback the signal as measured is small, enhancing the linearity of the magnetometer.

As noted earlier, the block diagram reflects the approach used for TRACERS MAG. For MMS, while the basic drive-sense-feedback principles apply, most of the drive-sense-feedback loop was implemented using analog rather than digital components. For this reason, the UCLA magnetometer flown on MMS was referred to as the Analog Fluxgate magnetometer (AFG). MMS also included a Digital Fluxgate magnetometer (DFG), provided by the Austrian Space Research Institute (Institute für Weltraumforschung, IWF). The DFG used an Application Specific Integrated Circuit (ASIC) that included sigma-delta modulators to perform analog to digital conversion (Magnes et al. 2008). While Magnes et al. specifically address an ASIC implementation, they also note that the approach could be implemented using an FPGA. At UCLA an FPGA version of the sigma-delta was developed by David Pierce and Kathryn Hector (formerly Rowe), which they referred to as the Pierce-Rowe Magnetometer (PRM). A multi-stack module that combined the FPGA and the associated electronics boards was built by 3DPlus, and flown with an ST5-style sensor on ELFIN-L (Shprits et al. 2018) and on the two ELFIN spacecraft (Angelopoulos et al. 2020). In parallel with this effort a more traditional circuit board design with a board-mounted FPGA, rather than a multi-stack module, was used for the InSight magnetometer (Banfield et al. 2019). It is this version of the electronics that provides the flight heritage for the TRACERS MAG electronics.

### Design and Fabrication

**Fig. 3:**
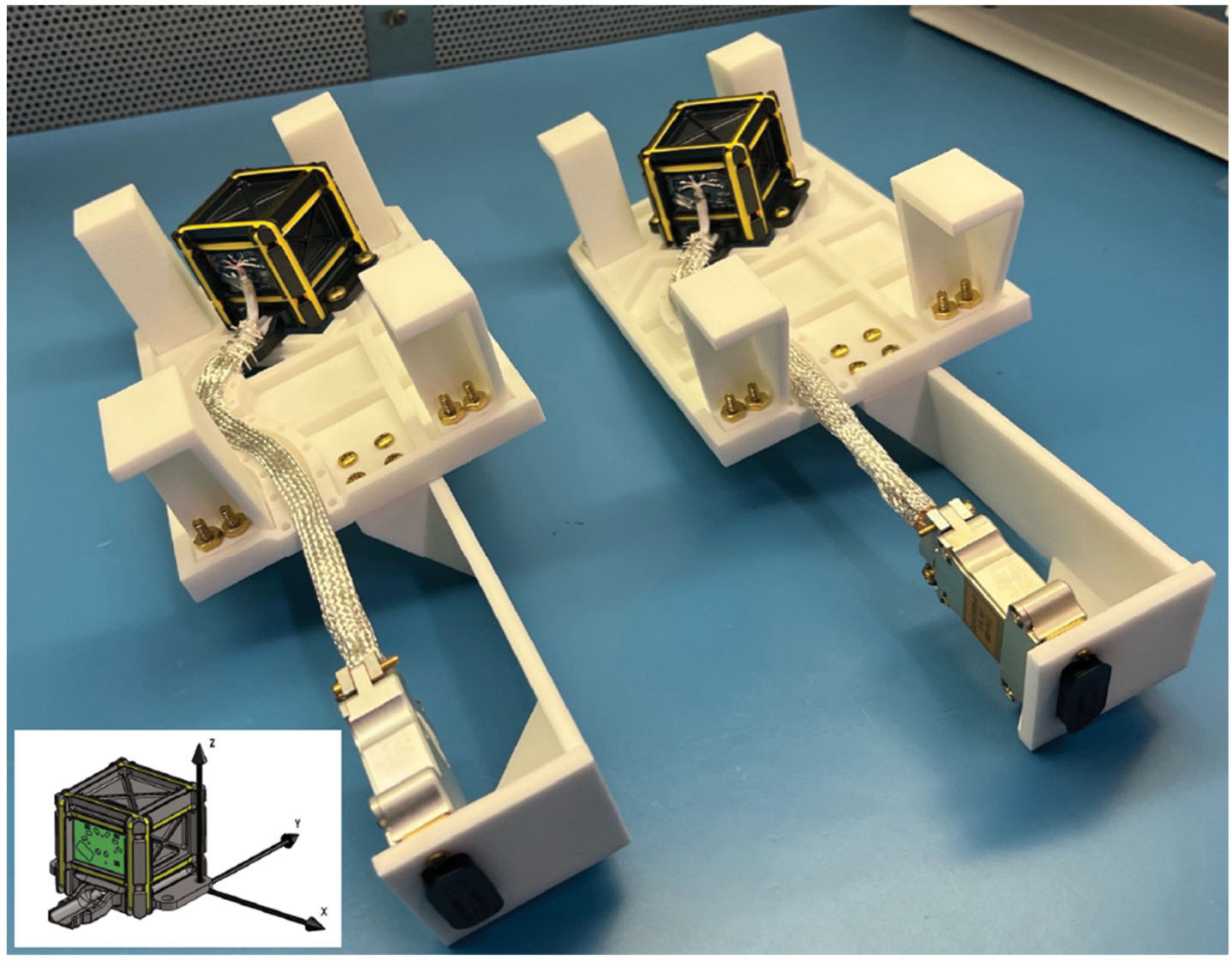
The two TRACERS MAG flight sensors in their handling fixtures (one for each spacecraft)

**Fig. 4:**
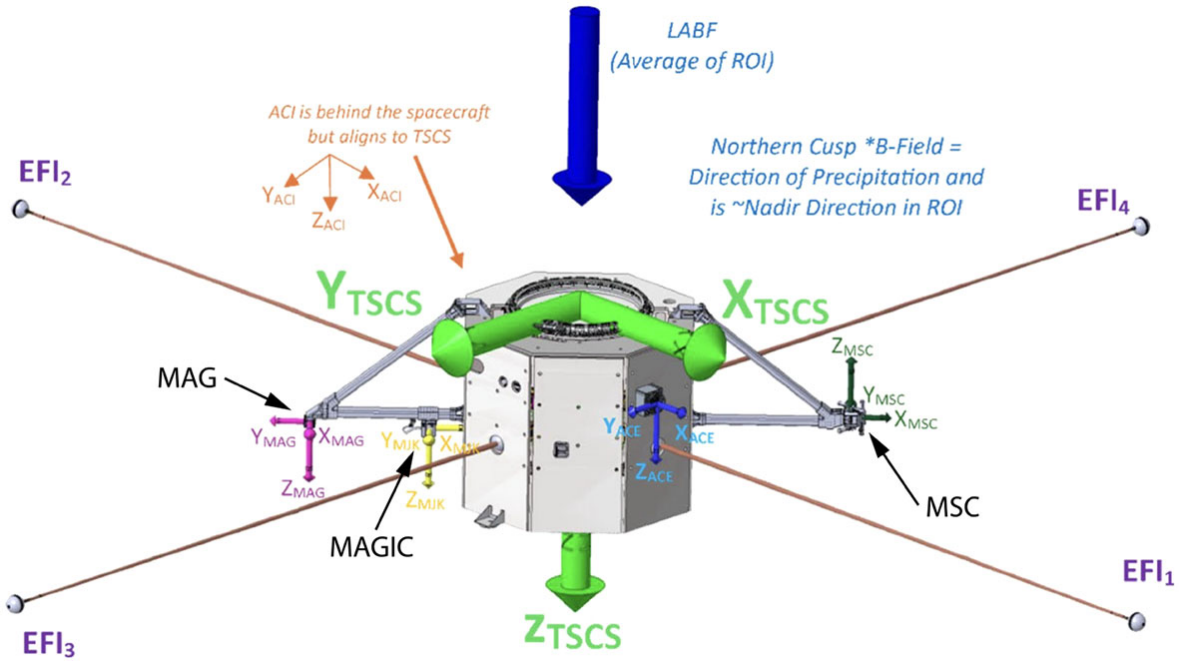
Drawing of one of the TRACERS spacecraft showing the fixed bracket assembly that replaced the deployable boom

Turning now to the design and fabrication of the TRACERS MAG, the sensor design was modified so as to be mounted to a base plate on a boom. The two flight sensors are shown in Fig. 3. They are fastened to handling fixtures that reflect the orientation of the sensors on the originally planned deployable boom. The 45-degree rotation in the handling fixture was such that the $x$- and $y$-axes of the sensor were aligned with the Electric Field Instrument (EFI) stacers (the inset in the figure shows the $x$- $y$- and $z$-axes of the MAG sensor) but, because of concerns with vibration loads, the boom was replaced with a fixed bracket assembly late in Phase C of the TRACERS project, see Fig. 4 (reproduced from Fig. 14 of Miles et al. 2025a).

As noted above, Fig. 4 shows a sketch of one of the two TRACERS spacecraft (after Fig. 14 of Miles et al. 2025a). The figure shows the spacecraft in its nominal on-orbit configuration, with the spin-axis pointing down in the northern hemisphere. The two brackets are the structures at the left and right of the spacecraft body. On the left-hand bracket we see MAG at the end, with the technology demonstration MAGIC magnetometer (Miles et al. 2025c) inboard of MAG. The Magnetic Search Coil (MSC, Hospodarsky et al. 2025) is mounted on the right-hand bracket. Both MAG and MSC are 70 cm from the surface of the spacecraft, while MAGIC is 20 cm away from the surface of the spacecraft (MAG and MAGIC are separated by 50 cm). The figure also shows the Electric Field Instrument (EFI) stacers, and the location of the Analyzer for Cusp Electrons (ACE). The Analyzer for Cusp Ions (ACI) is behind the spacecraft in this viewing angle.

In order to reduce any additional design requirements for the bracket, the fastening of MAG to the bracket has been simplified, and the MAG $y$-axis is parallel to the arm of bracket pointing away from foot of the bracket, i.e., to the left in Fig. 4. Consequently, the MAG sensor axes in the nominal spin plane are no longer aligned with the EFI stacers. Note that the $x$-axis of the TRACERS Satellite Coordinate System (TSCS) is along the EFI_1_ stacer. Because of this change in alignment the MAG and EFI measurement axes are no longer aligned in the spin plane, and a direct comparison of one component of the electric field with the corresponding magnetic field that is perpendicular to that EFI component (e.g., to estimate Poynting flux) is no longer possible without additional processing. The MAG data must be rotated by 45°, resulting in the magnetic field that is aligned with the EFI axes being a combination of both spin-plane components.

**Fig. 5:**
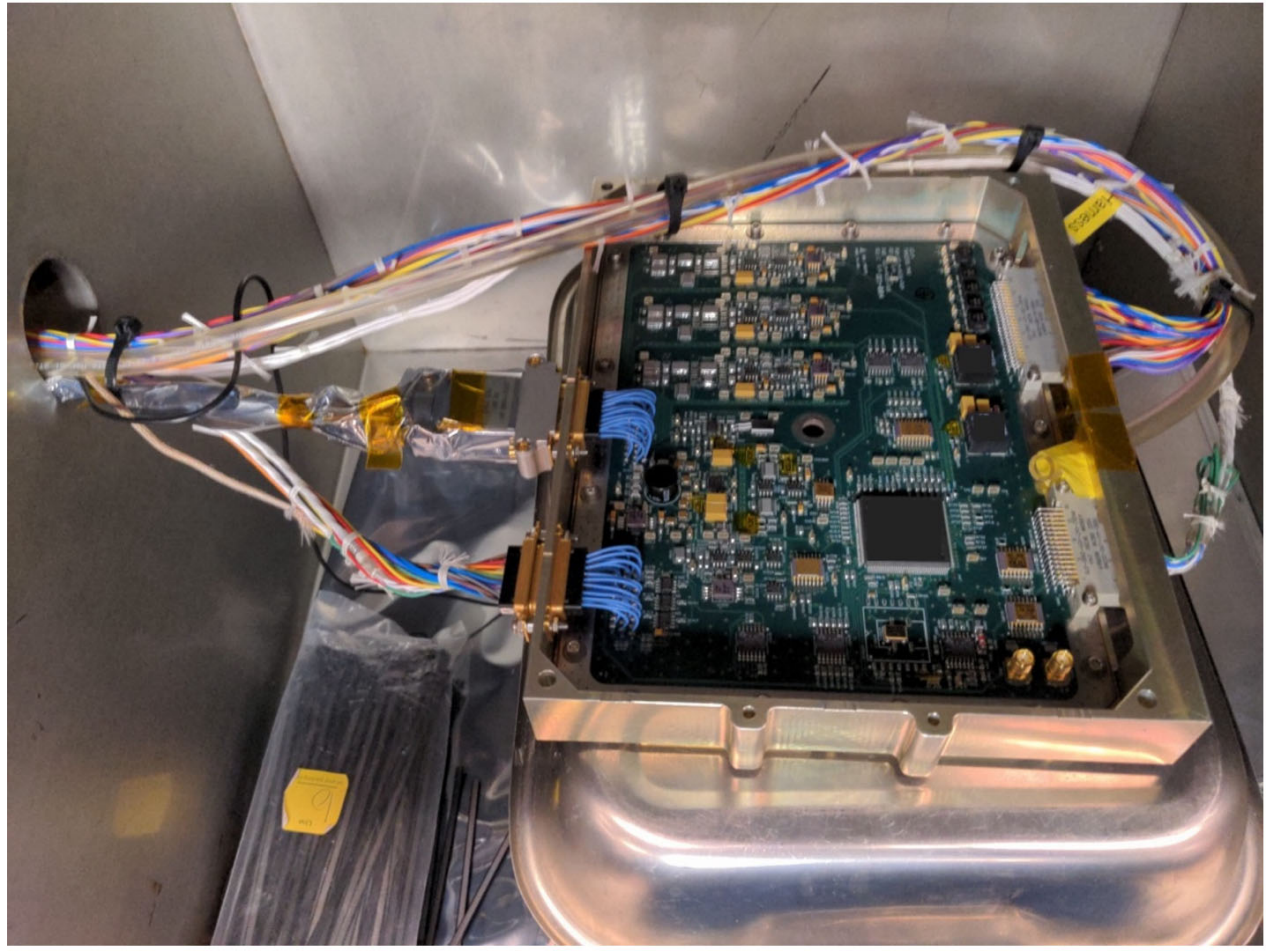
Populated Flight Model 1 (FM01) electronics board in the Tenney thermal test chamber at UCLA

In closing this section, Fig. 5 shows a photograph of the Flight Model 1 (FM01) MAG electronics board undergoing testing in the Tenney thermal test chamber at UCLA. The boards and sensors for both flight models have also been tested extensively after delivery to the University of Iowa. The results of some of these tests conducted at UCLA and the Univ. of Iowa will be discussed in greater detail in the next section, where we address verification of the requirements listed in Table 1 as well as the determination of the pre-launch MAG calibration parameters.

## Requirements Verification and Pre-Launch Calibration

As part of the MAG instrument delivery several different tests were performed to verify that the instrument meets the requirements. As noted in Table 1, some of these requirements were directly related to Science Objective 3 (SO3), with MAG providing magnetic field measurements associated with currents flowing into and out of the topside ionosphere. We will summarize the specific tests that verified the SO3-based requirements.

### Offset Determination

First, we discuss the offset determination that helps to satisfy the accuracy requirement. As noted in the previous discussion of Table 1, the 100 nT accuracy requirement was essentially an estimate of what would ensure that MAG could make the required measurements for SO3. We already noted in the previous section that, as the requirements were refined, the choice was made that 25 nT of the 100 nT would be specifically assigned to MAG, basically accounting for possible perming of the permeable material that is part of a fluxgate magnetometer. It was then recognized that the 25 nT should apply for each axis, so an additional requirement of 43 nT (=$\sqrt{\phantom{|}}$(3×25^2^) was levied on MAG.

**Fig. 6:**
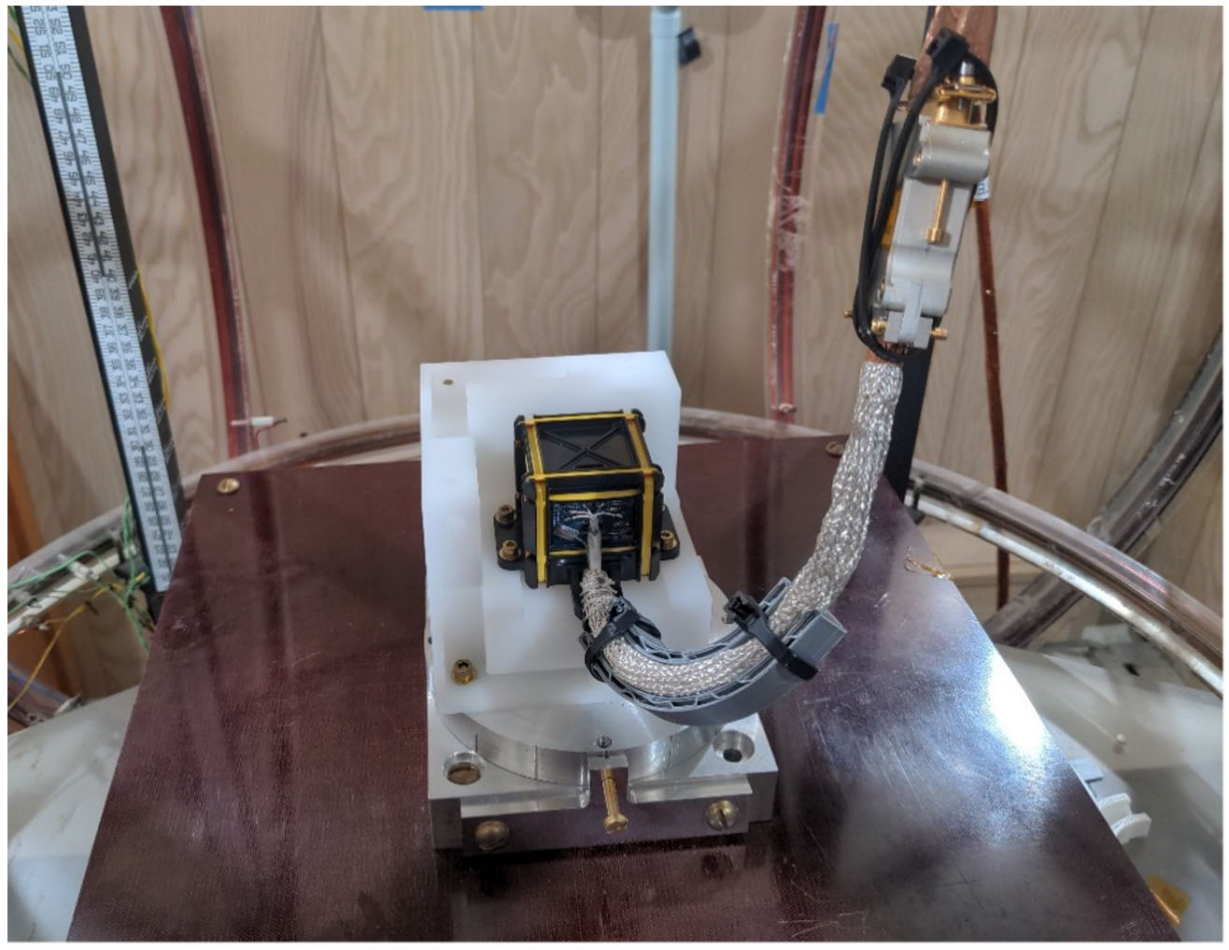
FM01 flight sensor in UCLA’s mu-house calibration facility

The MAG offsets were determined by a test performed in UCLA’s magnetically screened mu-house. The mu-house has a set of coils that can apply a magnetic field to be measured by a test instrument. This is shown in Fig. 6, which also includes the FM01 sensor mounted to a fixture that is in turn mounted to an aluminum turntable (the turntable is being held at a fixed angle by the bolt towards the bottom of the figure). For this particular test the sensor $z$-axis is vertical, and the sensor rotates about this axis as the turntable is rotated. A stimulus is provided in the horizontal direction. The turntable is rotated by hand.

**Fig. 7:**
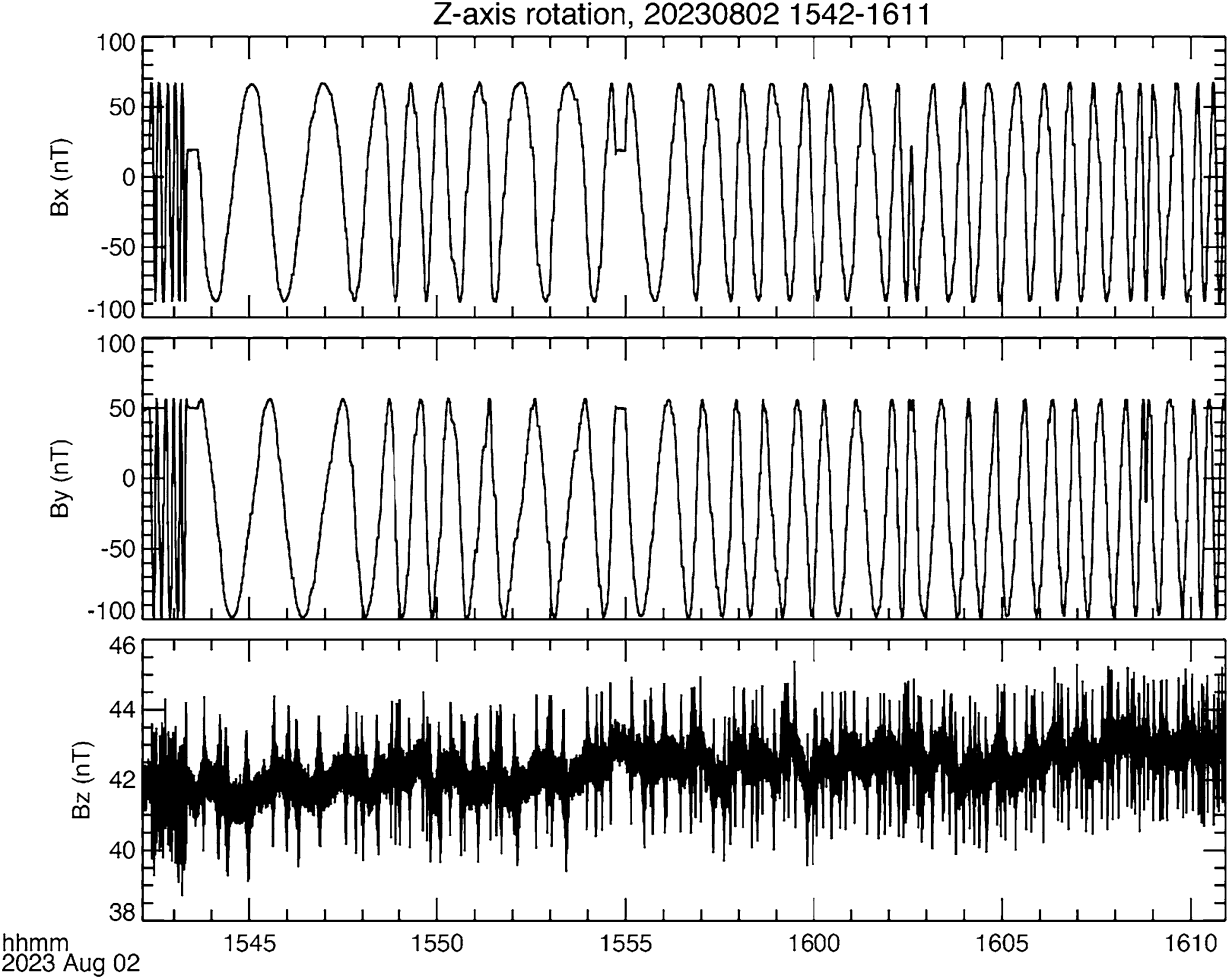
Data acquired when the sensor is rotated about the $z$-axis

Figure 7 shows the data acquired during this test. The irregularity in the period and phase of the rotation is because the turntable is rotated by hand. As expected, the rotation of the sensor is mainly seen in the $x$- and $y$-axes. Close inspection of the $z$-axis indicates a low amplitude signal that is roughly in anti-phase with the magnetic field measured by the $x$-axis. This most clearly seen around 1545 UT. This axis also has spikes of order 4 nT peak to peak. The exact cause of these is not known but may be associated with having to adjust the harness as the turntable is rotated.

In Fig. 7 for the signals observed in the $x$- and $y$-axes the nominal peak to peak is around 150 nT, but the signals are offset relative to zero. This offset is the desired parameter to be derived from the test, given by $B_{\mathrm{off}} = {\left ( B_{\mathrm{max}} + B_{\mathrm{min}} \right )} / {2}$. In order to give an error estimate $B_{\mathrm{off}}$ was determined separately for 10 rotations of the turntable. For FM01 the sensor was rotated about all three axes (providing offset determination twice for each axis), while for expediency the FM02 sensor was rotated about only the $x$- and $y$-axes (providing a single $x$- and $y$-offset determination and two $z$-offset determinations). We will not include figures for the other two sequence of rotations of FM01 or the two for FM02 since the data are very similar. Instead, we will summarize the results here, in Table 2.

**Table 2:** MAG offsets

Instrument	Axis	Offset (nT)	1-*σ* Error (nT)
FM01	*x*-axis	-10.856	± 0.082
	*y*-axis	-21.249	± 0.057
	*z*-axis	21.048	± 0.067
	magnitude	31.818	± 0.120
FM02	*x*-axis	-17.317	± 0.067
	*y*-axis	-17.888	± 0.027
	*z*-axis	13.249	± 0.050
	magnitude	28.202	± 0.088

Table 2 includes the magnitude of the offsets. Both instruments meet the 25 nT per component and the 43 nT magnitude requirements. Given the 1-$\sigma $ error, we will only specify the offsets to one decimal place when we summarize the pre-launch calibration results. It should also be remembered that the pre-launch calibration is a baseline for the improvements obtained through on-orbit calibration. In addition, the sign convention is such that the offsets given in Table 2 should be subtracted from the measured fields to provide corrected data. To further elaborate on this, in Fig. 7 the data for both $x$- and $y$- axes are shifted in the negative direction, corresponding to a negative offset. Adding minus the offset, i.e., +10.856 nT and +21.249 nT, respectively, centers the data.

### Post Delivery Tests

**Fig. 8:**
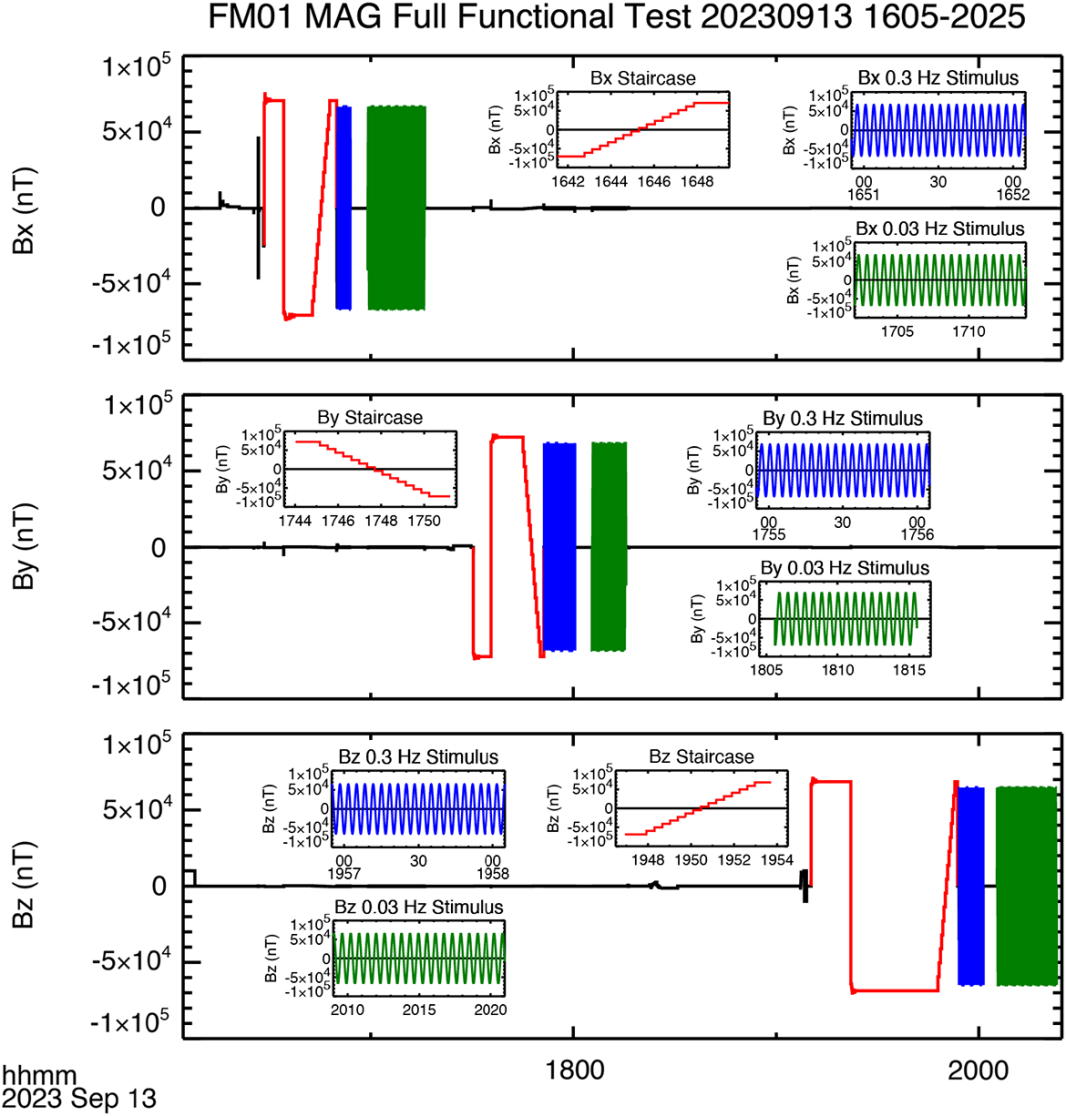
Data acquired during the Full Functional Test of FM01

**Fig. 9:**
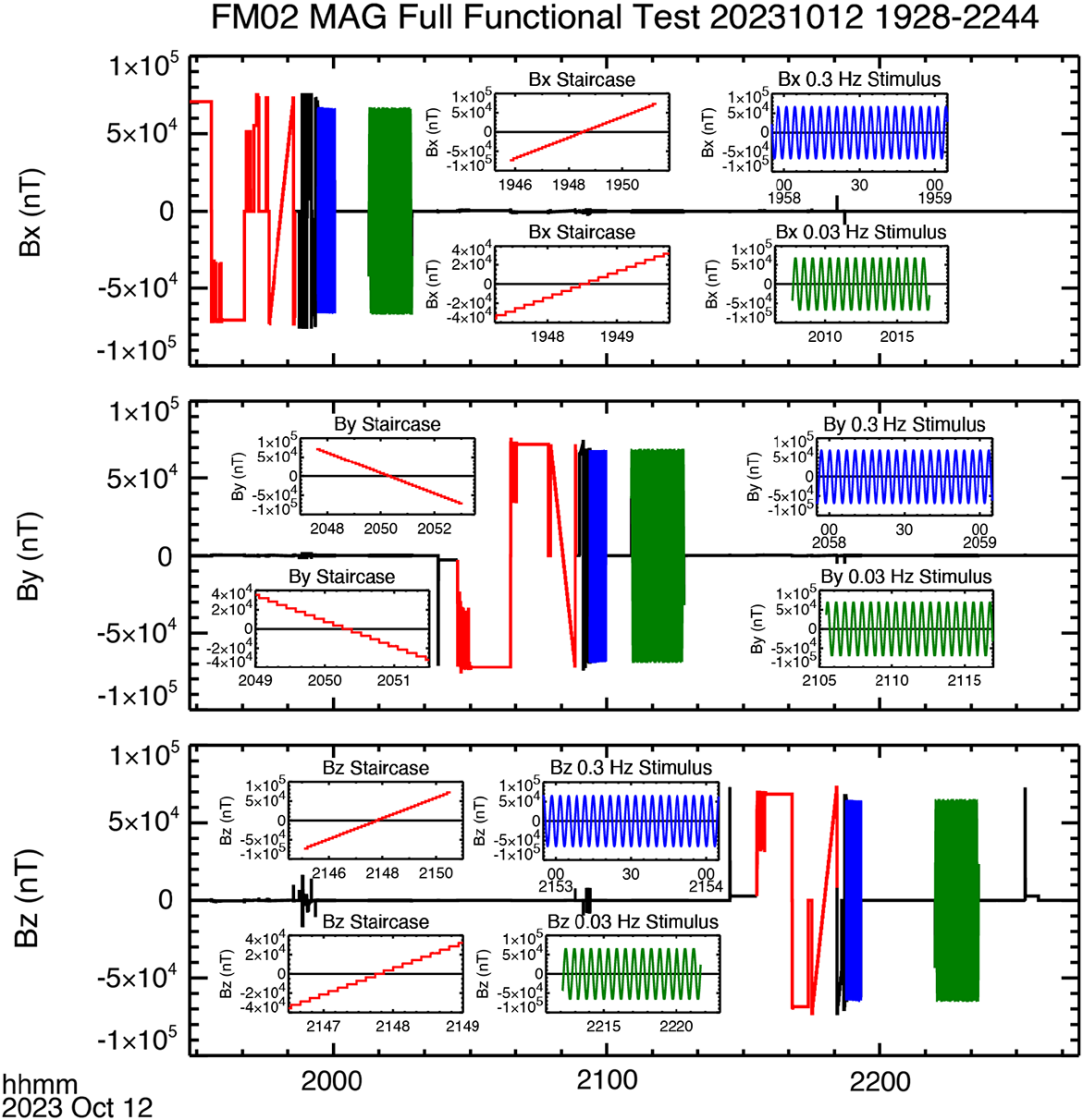
Data acquired during the Full Functional Test of FM02

After delivery of the flight units to the University of Iowa the flight boards were conformally coated, and a Full Functional Test was performed to address several of the instrument requirements. The data acquired for the FM01 test are shown in Fig. 8, and in Fig. 9 for FM02.

#### Dynamic Range

In Figs. 8 and 9 data for the complete duration of the test are shown, with inserts showing zoomed-in parts of the time series. Using the top panel of Fig. 8 as an example, the data shown in red are data used to determine the dynamic range for the specific sensor axis. The procedure to do this involves increasing (or decreasing for negative fields) the applied field until an out-of-range flag is tripped. The applied field is then reduced slightly and used to for the beginning and end of the “staircase” that can be seen at the end of the sequence of red data points. Note that, since the staircase data are hard to resolve in the full time series, shorter timespans of data are shown as inserts. In Fig. 8 each of these inserts covers 12 minutes of data. In Fig. 9 the staircase intervals were shorter and the first insert for each component (placed in the upper half of each panel) covers 6.5 minutes. A shorter interval of 2.5 minutes, which doesn’t cover the entire ramp, is included in the lower half of each panel to better resolve the steps in the data. The maximum and minimum values of the staircase are used as an estimate for the dynamic range of the instrument. Table 3 gives the estimated minimum and maximum values for both flight models, based on the staircase data shown in Figs. 8 and 9. The dynamic range exceeds ± 68,000 nT for all axes on both instruments.

**Table 3:** MAG dynamic range

Instrument	Axis	Range (nT)	
		Minimum	Maximum
FM01	*x*-axis	-70,640	70,585
	*y*-axis	-72,172	72,173
	*z*-axis	-68,591	68,596
FM02	*x*-axis	-73,068	73,056
	*y*-axis	-71,214	71,109
	*z*-axis	-72,888	72,927

#### Alignment

Following the staircase there is a sequence of blue and then green data points in each of the panels in Figs. 8 and 9. The panels inset to the figure show a high temporal resolution plot of these data. The data correspond to a 0.3 Hz and 0.03 Hz frequency stimulus. The purpose here was to verify that the magnetometer has no issues with acquiring data at both high and low spacecraft spin periods, ∼20 and ∼2 rpm, respectively (the test actually corresponds to 18 and 1.8 rpm). The expected on-orbit spin period is 10 rpm.

We shall use these data for a different purpose. Around 2100 UT the sine wave stimulus is applied to the $y$-axis. Careful inspection of the $x$-axis data acquired at this time indicates that the stimulus applied to the $y$-axis is also being sensed by the $x$-axis. We can therefore use these data to estimate the relative angles between the two axes. In particular, taking the ratio of the peak to peak amplitude of the $x$-axis response to the peak to peak amplitude of $y$-axis stimulus we get the cosine of the angle of the sense $x$-axis relative to the direction of the external stimulus. It is worth noting, that since this angle is nearly 90° the misalignment between the sensor $y$-axis and the applied stimulus is a small correction, of the order $1- \cos \delta $, where $\delta $ is the misalignment of the sense $y$-axis and the stimulus. It should be noted that the derived angle is the angle between the stimulus and the sense $x$-axis. To make this clear, we shall assume that the misalignment between the stimulus and the sensor $y$-axis in the plane defined by the $x$- and $y$-axis of the sensor is $\theta _{ys}$, and the corresponding angle for the $x$-axis is $\theta _{xs}$, and, further, that the elevation (out of sensor $x$-$y$ plane) angle of the stimulus is $\delta $. In defining these angles the subscript “s” indicates that the angle is relative to the in-plane stimulus. For each sensor axis the measured field corresponds to $B_{xs} = B_{s} \cos \delta \cos \theta _{xs}$ and $B_{ys} = B_{s} \cos \delta \cos \theta _{ys}$. Noting that $\theta _{ys}$ is expected to be small and $\theta _{xs}$ close to 90°, then ${B_{xs}} / {B_{ys}} = {\cos \theta _{xs}} / {\cos \theta _{ys}} =- {\sin \left ( \theta _{xs} - {\pi} / {2} \right )} / {\cos \theta _{ys}} \approx \left ( \theta _{xs} - {\pi} / {2} \right ) \left ( 1+ {\theta _{ys}^{2}} / {2} \right )$ (where the angles are expressed in radians). The error introduced by the misalignment of the sensor and the stimulus ($\theta _{ys}$) is small, a second order correction to $\left ( \theta _{xs} - {\pi} / {2} \right )$, which itself is also small ($\theta _{xs} \approx 90$°). But this also emphasizes that the derived angle is relative to the applied stimulus, it does not correspond to the angle between the sense $x$-axis and the sensor body $y$-axis as defined in Fig. 2.

The misalignment angles as determined by analyzing the response to the sine wave stimulus are summarized in Table 4. For ease of readability the angles have had 90° subtracted. For example, for FM01, $\theta _{yx} =90.181$°. It should be noted that the magnitude of the angles in Table 4 is generally small, less than 0.5°. Most are < 0.2°. It is therefore highly unlikely that the misalignment between the stimulus and sensor axes is a significant source of error. The angles in Table 4 consequently provide a good initial estimate for the off-diagonal terms in the coupling matrix.

**Table 4:** Misalignment angles

FM01				
$B_{\mathrm{x}}$ Stimulus	$B_{\mathrm{y}}$ Response	$B_{\mathrm{z}}$ Response	$\theta _{yx} -90$°	$\theta _{zx} -90$°
66,553 nT	-210.41 nT	-206.85 nT	0.181°	0.178°
$B_{\mathrm{y}}$ Stimulus	$B_{\mathrm{x}}$ Response	$B_{\mathrm{z}}$ Response	$\theta _{xy} -90$°	$\theta _{zy} -90$°
68,073 nT	-494.86 nT	60.455 nT	0.417°	-0.051°
$B_{\mathrm{z}}$ Stimulus	$B_{\mathrm{x}}$ Response	$B_{\mathrm{y}}$ Response	$\theta _{xz} -90$°	$\theta _{yz} -90$°
64,654 nT	54.154 nT	-144.90 nT	-0.048°	0.128°
FM02				
$B_{\mathrm{x}}$ Stimulus	$B_{\mathrm{y}}$ Response	$B_{\mathrm{z}}$ Response	$\theta _{yx} -90$°	$\theta _{zx} -90$°
66,102 nT	-282.80 nT	184.12 nT	0.245°	-0.160°
$B_{\mathrm{y}}$ Stimulus	$B_{\mathrm{x}}$ Response	$B_{\mathrm{z}}$ Response	$\theta _{xy} -90$°	$\theta _{zy} -90$°
68,258 nT	-423.10 nT	-80.242 nT	0.355°	0.067°
$B_{\mathrm{z}}$ Stimulus	$B_{\mathrm{x}}$ Response	$B_{\mathrm{y}}$ Response	$\theta _{xz} -90$°	$\theta _{yz} -90$°
64,397 nT	-30.255 nT	218.44 nT	0.027°	-0.194°

### Instrument Noise Level

**Fig. 10:**
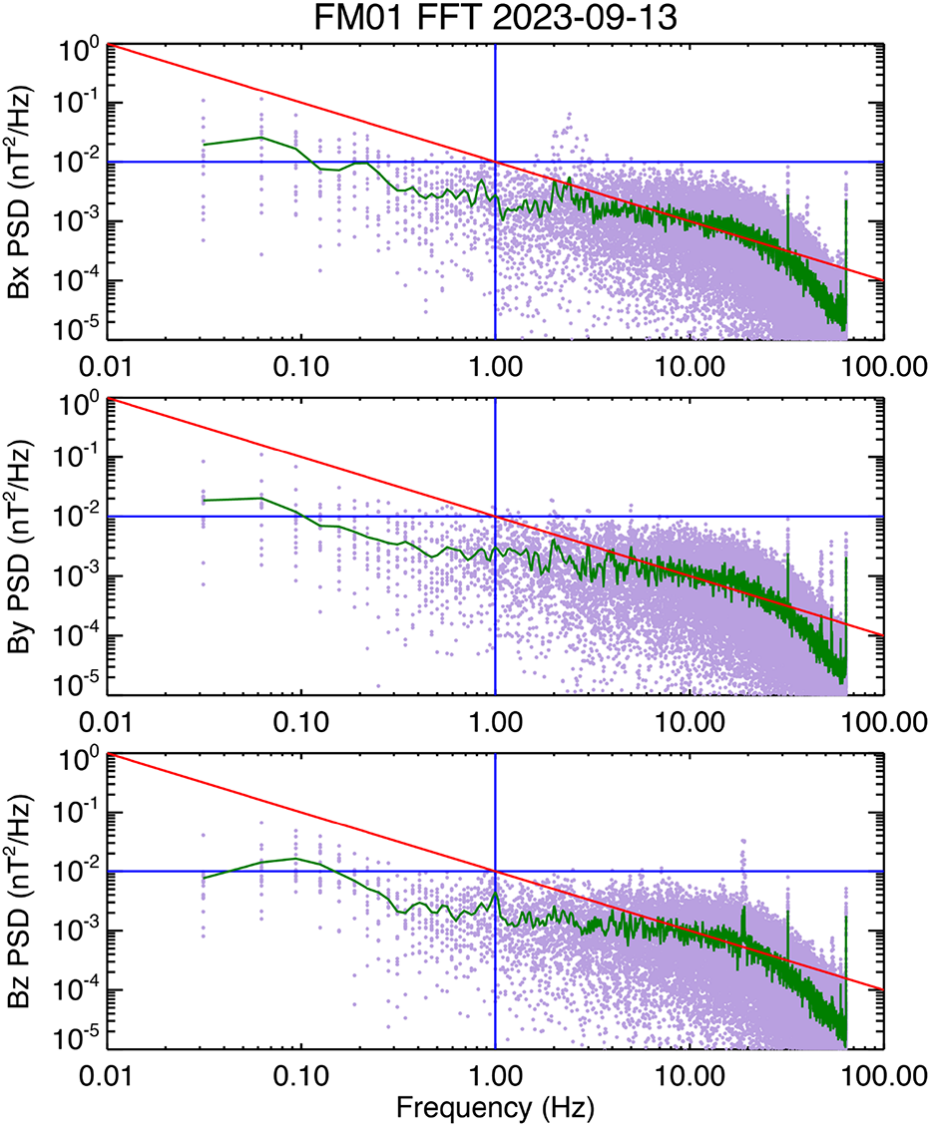
Noise spectra for FM01

We now have sufficient information to generate a pre-launch coupling matrix and offsets that can be used as a baseline for post-launch on-orbit calibration. Before doing that, however, we will address the last remaining requirement listed in Table 1, which is the instrument noise level. For FM01 we used data acquired during the staircase intervals as that allows us to explore noise levels across the full dynamic range of the instrument. Figure 10 shows the noise spectra for FM01. The spectra are plots of Power Spectral Density (PSD) in units of nT^2^/Hz as a function of frequency (Hz). In generating the noise spectra, data from each flat portion of the staircase interval are selected. The selection is checked to ensure that the ramped part of the data is not included. The data intervals are then detrended and zero-padded to make 128x32 data point samples. For Fig. 10, 16 individual spectra are generated, with the PSD at each frequency given by the dots with the low-saturation magenta color. A 16-point average is calculated for each frequency in the PSD, and this average is indicated by the green line. The data are 128 sps, and spectra cut off at the 64 Hz Nyquist frequency. The red line has a slope of 1/$f$ and passes through 0.01 nT^2^/Hz at 1 Hz, where the blue lines cross. The slope of 1/$f$ is a characteristic of the fluxgate magnetometer noise, at least for frequencies at or below 1 Hz.

Based on the average PSD, the FM01 MAG clearly meets the requirement given in Table 1, although the noise level does approach or cross the 1/f line around 10 Hz and higher frequencies. Strictly speaking the requirement does not specify the frequency dependence of the noise. Nevertheless, the flattening of the spectrum does reduce the MAG sensitivity to higher frequencies. We also note that some of the noise may be environmental, since there is a clear indication of 60 Hz noise. It is not clear if the other peaks seen in the spectra above 1 Hz are also environmental, or a residual of what is known as pattern noise. The TRACERS MAG FPGA code has been modified to reduce this as much as possible. The PSDs also indicate a reduction in the instrument response above 20 Hz. This is a result of the filtering in the MAG electronics that reduces the internal 8192 sps high-rate data to 128 sps (see Fig. 2).

While flattening of the noise spectra above 1 Hz suggests that the MAG may be less sensitive, it still responds to waves up to at least 10 Hz. Nevertheless, there is a planned Level 3 (L3) data product that can help address the flattening of the noise spectra, as well as any remanent of pattern noise. This L3 data product merges MAG and MSC data (Miles et al. 2025a). As noted by Hospodarsky et al. (2025), the MSC sensitivity drops off below 10 Hz. Given the flattening of the MAG noise spectra occurs above 1 Hz, the cross-over frequency of the merged L3 data product is expected to lie somewhere between 1 Hz and 10 Hz, with MAG data being the primary contributor to the merged product below 1 Hz, and MSC primary above 10 Hz. These frequencies are nominal at this stage, and the exact details of the algorithm that will be used to generate the L3 product will be determined based on MAG and MSC data acquired on orbit. The L3 product will mitigate any data quality concerns associated with the MAG noise levels above 1 Hz.

**Fig. 11:**
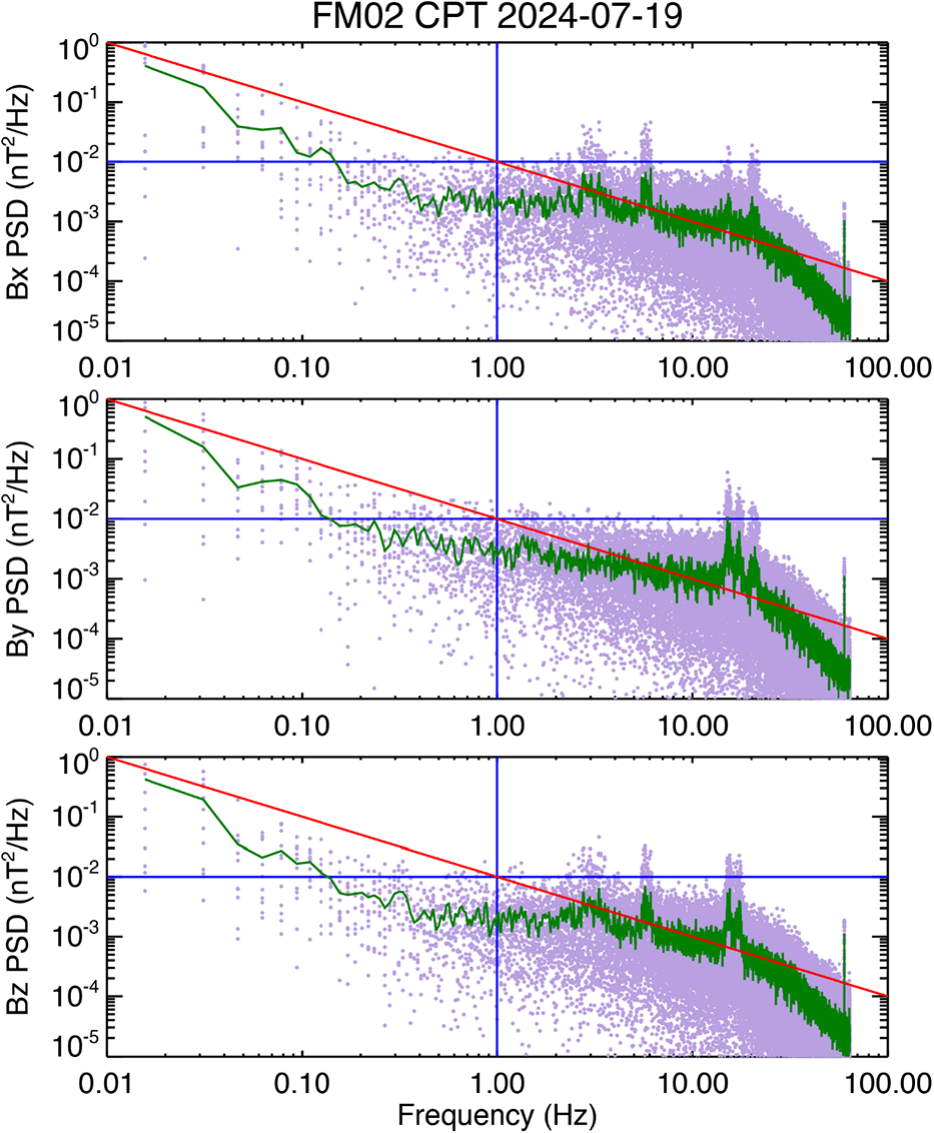
Noise spectra for FM02

The spectra derived use the staircase data for FM02 are somewhat noisier than FM01, and the $y$-axis slightly exceeded the 100 pT/$\sqrt{\phantom{|}}$Hz (0.01 nT^2^/Hz) at 1 Hz requirement. This may have been a consequence of leakage of the pulse per second synchronization signal that MAG provides to MAGIC. However, data from a subsequent FM02 Comprehensive Performance Test (CPT) had quieter background noise since the electronics board was integrated into the Master Electronics Box (MEB). This was a test where a chirp signal was applied to MAG, but we use 11 quiet data intervals excluding the chirps to generate PSD plots for FM02, shown in Fig. 11. In this case we used 128×64 detrended and zero-padded data points per FFT. The time intervals for the FFTs were chosen to avoid the intervals where a chirp signal was applied, resulting in one of the FFTs using 128×48 points, zero-padded to the same length as the other FFTs.

The data acquired during the CPT were in raw counts. To convert the counts to nT we used the gain factors used by the Science Operations Center (SOC) to generate MAG L1b data from the raw counts in the L1a data. Pre-launch calibration tests provided the preliminary gains that in turn were provided to the SOC by co-author Eric Orrill for implementation in the SOC software (Chris Piker, personal communication) and are summarized in Table 5. The final gain factors will be adjusted through on-orbit calibration. Because the gains will be further adjusted through on-orbit we refer to the resultant L1b data as being in “pseudo-nT”.

**Table 5:** MAG counts to pseudo-nT conversion factors

Sensor Axis	Counts to pseudo-nT
*x*-axis	0.008928883
*y*-axis	0.009201682
*z*-axis	0.008705114

The conversion factors in Table 5 are the same for both flight models. These conversion factors allow quicklook plots generated from L1b data to be scaled so that the plotted data are close to actual nT, sufficient for the primary purpose of such plots to both indicate the health of the instrument and provide a means for estimating if the ROI has scientific significance. In addition, by using the conversion factors in Table 5 as the starting point for the on-orbit calibration process, we expect the leading diagonal terms in the coupling matrix to be close to unity with the off-diagonal terms essentially corresponding to a misalignment angle in radians. We also note that these counts to pseudo-nT factors may be updated after launch, as a result of the on-orbit calibration during commissioning.

Returning to Fig. 11, the FM02 noise spectra are similar to the FM01 noise spectra. For both instruments we again note the presence of 60 Hz noise, as well as spikes at other frequencies. Since natural noise levels can be very low in space, once data are acquired on orbit we can determine if we need to filter the data to remove any remaining spikes in the L2 data. The merged MAG/MSC L3 data product will also take care of this issue.

### Pre-Launch Coupling Matrix and Offset Vector

The last topic in this section is to define a preliminary coupling matrix $\mathbf{M}_{p}$ and offset vector ($\mathbf{O}_{p}$) using the angles reported in Table 4 and the offsets in Table 2. The preliminary magnetic field is given by $\mathbf{B}_{p} = \mathbf{M}_{p} \boldsymbol{\cdot} \mathbf{B}_{m} - \mathbf{O}_{p}$, where $\mathbf{B}_{m}$ is the magnetic field as measured by the magnetometer using the counts to pseudo-nT conversion factors. We have used the subscript “$p$” to make it clear that the coupling matrix and offsets presented here are the starting point for MAG calibration. The post-launch on-orbit calibration will be used to update this preliminary pre-launch calibration.

For the preliminary coupling matrix there is no adjustment to the gain factors, and the leading diagonal terms are unity. The off-diagonal terms are given by $- \cos \theta _{ij}$, where the angles $\theta _{ij}$ are given in Table 4. The preliminary coupling matrix and offsets are given below.

1 $$\begin{aligned} &\text{FM1:} \\ &\left ( \textstyle\begin{array}{c} B_{px}\\ B_{py}\\ B_{pz} \end{array}\displaystyle \right ) = \left ( \textstyle\begin{array}{c@{\quad}c@{\quad}c} 1 & \; 0.007278 & -0.000838\\ \; 0.003159 & 1 & \; 0.002234\\ \; 0.003107 & -0.000890 & 1 \end{array}\displaystyle \right ) \times \left ( \textstyle\begin{array}{c} B_{mx}\\ B_{my}\\ B_{mz} \end{array}\displaystyle \right ) - \left ( \textstyle\begin{array}{c} -10.8\\ -21.2\\ \; 21.0 \end{array}\displaystyle \right ) \end{aligned}$$

2 $$\begin{aligned} &\text{FM2:} \\ &\left ( \textstyle\begin{array}{c} B_{px}\\ B_{py}\\ B_{pz} \end{array}\displaystyle \right ) = \left ( \textstyle\begin{array}{c@{\quad}c@{\quad}c} 1 & \; 0.006196 & \; 0.000471\\ \; 0.004276 & 1 & -0.003386\\ -0.002793 & \; 0.001169 & 1 \end{array}\displaystyle \right ) \times \left ( \textstyle\begin{array}{c} B_{mx}\\ B_{my}\\ B_{mz} \end{array}\displaystyle \right )- \left ( \textstyle\begin{array}{c} -17.3\\ -17.9\\ \; 13.2 \end{array}\displaystyle \right ). \end{aligned}$$

## Post-Launch on-Orbit Calibration

The coupling matrix and offsets given at the end of the previous section will be refined once the TRACERS spacecraft are launched. Being in a low Earth orbit, the magnetometer measurements are dominated by Earth’s magnetic field, around 50,000 nT at high latitudes. The basic approach to on-orbit calibration is to adjust the coupling matrix so that the magnetometer measurements match Earth’s magnetic field at the TRACERS orbit as predicted by reference models such as the International Geomagnetic Reference Field (IGRF). The most recent version of the IGRF is version 14, whose 5-year cadence coefficients are definitive (DGRF) from 1945 to 2020, IGRF for 2025, and annual secular variation for 2025-2030 (see Alken et al. 2021 for a description of IGRF-13). This assumes that, in a statistical sense, the effects of the perturbation magnetic fields wrt to the background, which are of scientific interest to TRACERS, can be averaged out if sufficient number of orbits of data can be combined. The approach of trending the observed magnetic field against the model field was used on the FAST project. Some of the FAST data are shown in Fig. 1. By way of a consistency check, we note there is reasonable consistency between the magnetic and electric field measurements, suggesting that this approach works.

### Coupling Matrix and Offset Variability

**Fig. 12:**
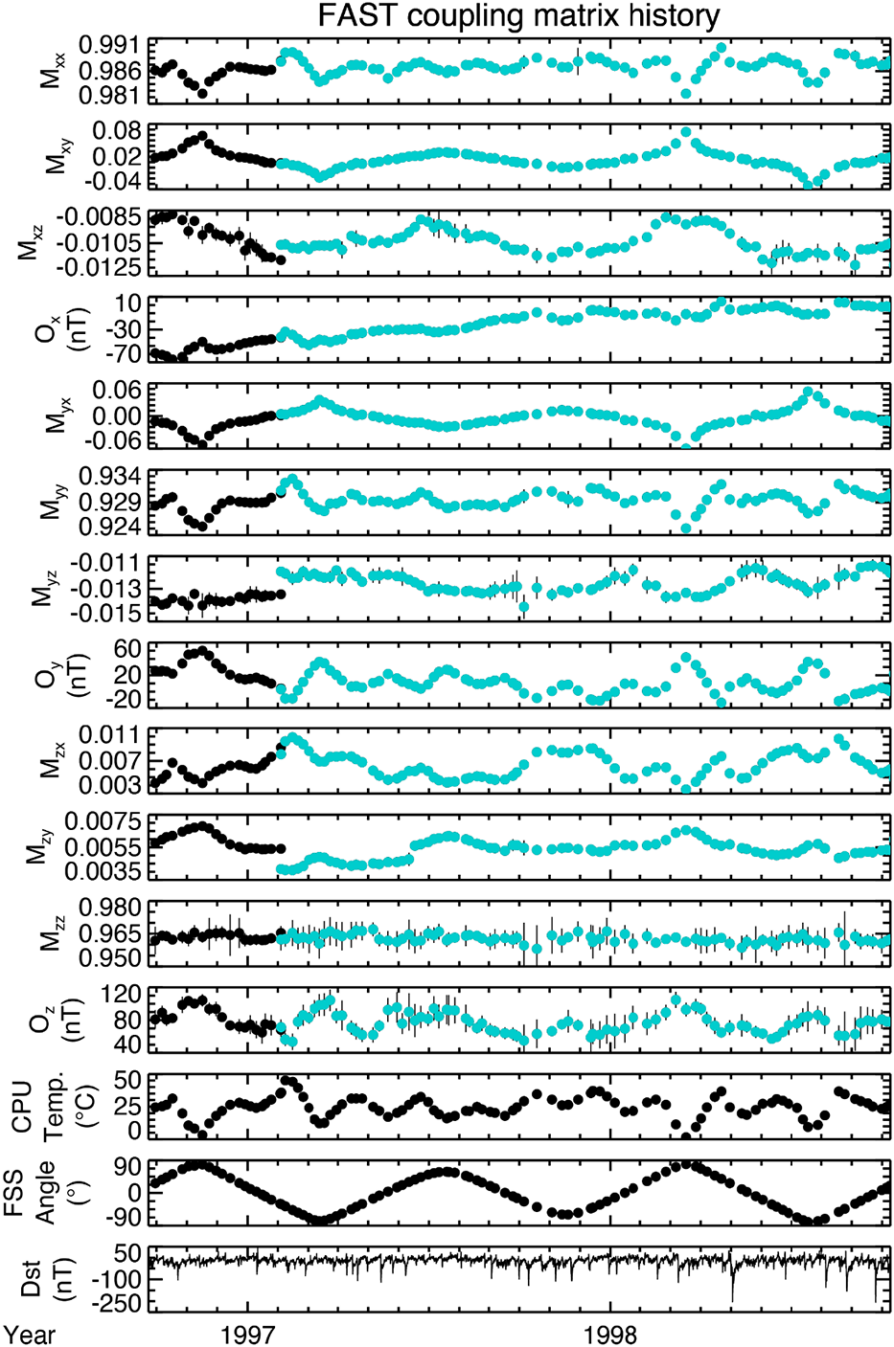
FAST coupling matrix and offsets

Figure 12 shows calibration data for the first two years of the FAST mission. Before going into details, the color change about 1 month into 1997 corresponds to when one of the axial booms was deployed, resulting in a change of the spin axis direction relative to the body axes. The first four panels correspond to the top row of Eq. (1) in the previous section. $M_{xx}$ is $xx$-component of the coupling matrix $\mathbf{M}$ (we have dropped the subscript “$p$” as this is now the flight version of the coupling matrix), with $M_{xy}$ below this, and $M_{xz}$ plotted in the third panel. $O_{x}$ is the $x$-component of the offset vector $\mathbf{O}$. The next four panels correspond to the second row of Eq. (1), i.e., to $M_{yj}$ and $O_{y}$, and the four below these to $M_{zj}$ and $O_{z}$. The remaining three rows show the elevation angle of the Sun as measured by the FAST Sun Sensor (FSS), the electronics board temperature, and the Dst index. The latter is used to identify days where the comparison with the model of the Earth’s magnetic field may be compromised by variability associated with changes in Dst, as well as other current systems that intensify during times of geomagnetic activity. The temperature and FSS angle are included as some of the variation in the calibration appears to be correlated with these.

The approach to on-orbit calibration involves three steps. The first is to perform high cadence spin-tone regression analysis (on FAST this was done with a two-spin cadence). This provides adjustments to the coupling matrix elements $M_{yy}$ ($y$-axis gain), $M_{yx}$ ($y$-axis orthogonalization), and $M_{zx}$ and $M_{zy}$ ($z$-axis alignment with the spin-axis), and the two spin-plane offsets $O_{x}$ and $O_{y}$. As part of the updates to the calibration parameters these are reduced to orbit averages. The next step is to perform linear regression analysis of the spin-plane measurements against the spin-axis measurement. This allows us to adjust the $x$- and $y$-axis so that they are in the spin plane (coupling matrix parameters $M_{xz}$ and $M_{yz}$). The final step is to perform regression analysis against the model field. This provides updates to the absolute gains ($M_{xx}$, $M_{yy}\ M_{zz}$), a spin-plane phase adjustment that results in a rotation of the spin-plane components of the coupling matrix, and the spin-axis offset ($O_{z}$). Although generated once per orbit, the data from the Earth field comparison are usually averaged over several days. The exact length of the average will be determined as part of the commissioning.

In general, the spin-plane gain adjustments ($M_{xx}$ and $M_{yy}$) show some correlation with the electronics temperature, as do some other parameters ($M_{zx}$, $O_{y}$, and $O_{z}$). A quick estimate, based on the data as plotted in Fig. 12 indicates $M_{xx}$ and $M_{yy}$ vary by 1% over 40 °C, $M_{zx}$ varies by 5 mrad over 40 °C, $O_{y}$ varies by 2 nT/°C, and $O_{z}$ varies by 1 nT/°C. From a pragmatic point of view, the main purpose in deriving these parameters is to generate the highest quality science data product for MAG.

The alignment of the spin-plane components, corresponding to $M_{xy}$ and $M_{yx}$, has a strong dependence on the elevation angle of the star sensor. This is because the FAST spacecraft spin axis and the body z-axis were misaligned because one of the radial wire booms failed to deploy fully (see Pankow et al. 2001 for a discussion of the booms used on FAST). For high elevation angles the time of the sun pulse will be early or late by a time corresponding to about 3.5 degrees (0.06 radians) of rotation. Since the spacecraft despun coordinate system uses the direction of the Sun, the magnetometer data have to be rotated by the spin-phase error for them to be aligned with the model field.

Of all the calibration parameters shown in Fig. 12, only one shows a roughly linear variation with time: the $x$-axis offset ($O_{x}$). This varies by about 3 nT/month.

A final remark concerning the data shown in Fig. 12 is the larger probable errors for the $z$-axis gain ($M_{zz}$) and $z$-axis offset ($O_{z}$) (the probable error is given by the vertical line behind each data point). The misalignment of the $z$-axis wrt the spin axis is usually well determined, as it manifests itself as a spin tone, but the gain and offset determination for the spin axis is less robust. The FAST spacecraft was flown in a reverse cartwheel orbit and the Earth’s magnetic field was close to the spin plane when the spacecraft was at auroral and polar latitudes. For TRACERS the spin-axis is nominally aligned with the background magnetic field in the ROI, making any uncertainties in the calibration for the $z$-axis less of a concern.

### Calibration Adjustments Using Spin-Fit Data

**Fig. 13:**
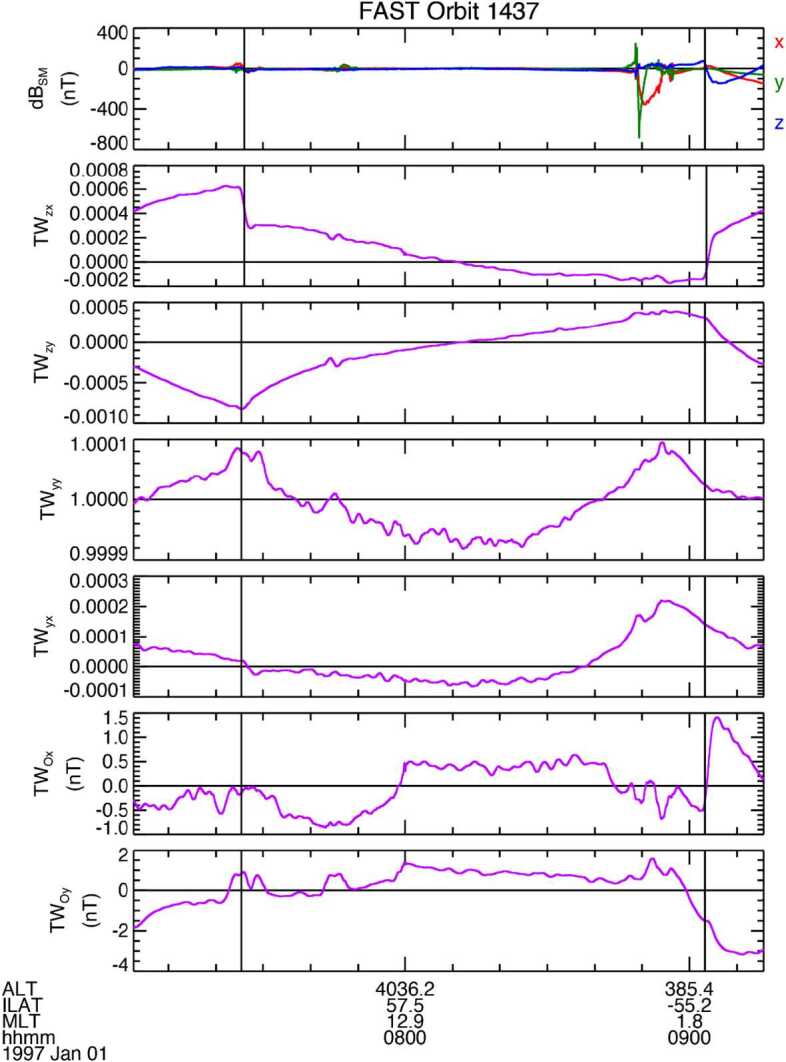
FAST calibration tweaker example

In addition to the calibration data shown in Fig. 12, high cadence calibration data were generated for FAST using two spin-period analysis of the correlation between different components. Data for one orbit are shown in Fig. 13. It should be noted that for FAST spinning, rather than despun, data were used. For other missions (e.g., MMS, Russell et al. 2016), the data are despun, which has the effect for the spin-plane sensors of making the offset be seen as a signal at the spin-tone. The decision to use spinning data for FAST was made because even over two spins (10 s) the spacecraft moved several kilometers, sufficient for the direction of the background field to change. Instead, the zero-crossing time of one of the spin plane components was used to define the period of a sine and cosine wave used for regression analysis.

For each magnetic field component the regression analysis returned the amplitude of the cosine and sine of the spin-phase dependent variation and a constant. The results from the regression analysis were used to adjust the phase and amplitude of the $y$-axis relative to the $x$-axis in the “tweaker” adjustments to the coupling matrix ($TW_{yx}$ and $TW_{yy}$, respectively) such that the $y$-axis was orthogonal to the $x$-axis. For the $x$- and $y$-axis the constant coefficients from the regression analysis provided adjustments to the spin-plane offsets ($TW_{Ox}$ and $TW_{Oy}$). Lastly, the spin-tone fits for the $z$-axis provided estimates of the projection angles of the magnetometer $z$-axis onto the spin-plane $x$- and $y$-axis ($TW_{zx}$ and $TW_{zy}$). The adjustments provided by the tweaker analysis resulted in the magnetic field components being orthogonal and little or no spin-tone in the despun magnetic field measurements.

The top panel of Fig. 13 shows the measured magnetic field in Solar Magnetic (SM) coordinates, with the remaining panels showing the tweaker adjustments to the coupling matrix and offsets. These are small, but clearly measurable. For the orbit shown the FAST spacecraft exited eclipse around 0725 UT and entered eclipse again around 0905 UT (eclipse exit and entry are indicated by the vertical lines). The spin-axis sensor misalignment with respect to the spin-axis changed over the orbit, with the initial and final alignment being the same (see TW_*zx*_ and TW_*zy*_). Elphic et al. (2001) attributed this to bending of the 2-m boom. As noted above, this was small, sub mrad. Furthermore, boom flexure will be less of a problem for TRACERS, given the switch to the bracket design shown in Fig. 4. There are also small changes in the gain adjustment used to make both spin-plane sensors report the same magnitude for the spinning field (TW_*yy*_) and be in quadrature (TW_*yx*_). The offsets (TW_*Ox*_ and TW_*Oy*_) also show variability.

Since most of the parameters in Fig. 13 are small it could be asked if such fine tuning is required. The approach taken with FAST is that if a change in alignment or offset could be characterized, then, through an abundance of caution, it should be removed.

In this section we have outlined the approach used for on-orbit calibration of the MAG data. We have not yet described how the on-orbit calibration is integrated with the generation of the L2 data products. This is discussed in the next section.

## Operation and Data Products

Post launch operations for MAG are relatively simple, in that the MAG is either powered on or powered off. The change in data rate from 128 sps in the ROI to 16 sps in the BOR is performed through commands sent to the CDPU in the MEB. The generation of Level 2 (L2) data files, on the other hand, is more complicated. This is because part of the process involves updating the calibration parameters. Indeed, it is likely that the creation of L2 will be a two-step process. MMS (Russell et al. 2016) used an intermediate step (L2 preliminary or L2pre) that used the latest calibration parameters available at the time to generate science data. These data were sufficiently useful for other instruments that could use less precise magnetic field measurements (e.g., to specify pitch angle) for their L2 product. All the instruments on MMS had the same latency requirement for delivering their L2 data, so the other instruments could not wait for magnetometer L2 data. The magnetometer team on MMS also used the L2pre data for the update to the calibration parameters (coupling matrix and offsets), rather than a lower-level data product. L2pre used the latest calibration parameters and any subsequent changes to the calibration parameters were expected to be of first order. MMS had a weekly magnetometer team conference (MagCon) where updates to the calibration were presented and approved. Once approved a new version of the calibration file was released.

For TRACERS the current plan is to also have a preliminary L2 data product. In a Technical Interchange Meeting held at the University of Iowa in May 2025 it was decided that this preliminary data product should remain with the instrument team and given the designation L1c. The L1c data will be the basis for updating the calibration, where orbit-by-orbit comparison with the Earth’s field will be used to generate data that are first order corrections to the pre-existing calibration data that are then used to propagate the calibration forward, again on a weekly cadence. For MMS the magnetometer instrument team involved people from many institutions, and the MagCon took on a more formal nature, occurring weekly via video conference. For TRACERS the process of verifying and updating the calibration will also require at least weekly meetings. More frequent meetings may be necessary if the calibration data are found to vary significantly. The meetings will not require multiple institutions to be involved, making the scheduling easier. The calibration data files will include all the parameters shown in Fig. 12.

For FAST the data cadence was several days, sometimes more than a week if Dst indicated significant geomagnetic activity. The actual cadence for TRACERS will depend both on the intrinsic variability of the calibration parameters themselves, and geomagnetic activity. For discussion purposes we assume the cadence will be weekly, but, again, the actual cadence will be determined after launch.

TRACERS MAG will also follow the approach developed for FAST, where spinning data are used to obtain on-orbit “tweaks” to a subset of the calibration data (Fig. 13). Since these data will have a cadence of the order a few spins, they will be included with the full calibration data generated on a roughly weekly basis as support data in the L2 files. These data are included mainly for purposes of traceability.

The L2 files will include magnetometer data in several different coordinate systems, as listed in Table 6. The text in parentheses after the variable name is given in lower-case to match the variable naming convention in the L2 files. While many of the coordinate systems are standard, some are specific to TRACERS. In particular, TSMAG (TRACERS MAG) and TSCS (TRACERS Spacecraft Coordinate System) are both spinning coordinate systems, fixed to the spacecraft. TSS is the despun TRACERS Spin Sun coordinate system. The definitions of all the coordinate systems listed in Table 6 are defined in Appendix A of Christopher et al. (2025). The IGRF-14 data have been included in the section labelled ephemeris data, as the IGRF is calculated using the spacecraft position in Geographic coordinates.

**Table 6:** Data included in MAG L2 files

Science Data	
Variable	Coordinate System
Full Vector Field (b)	TSMAG (TRACERS MAG)
	TSCS (TRACERS Spacecraft Coordinate System)
	TSS (TRACERS Spin Sun)
	GEI 2000 (Geocentric Equatorial Inertial J2000 Epoch)
Perturbation Field (deltab)	GEI2000
	GEO (Geographic)
	SM (Solar Magnetic)
	FAC (Field Aligned Coordinates)
	FVC (Field-Velocity Coordinates)

Ephemeris Data	
Variable	Coordinate System
Position (pos)	$\left \{ \begin{array}{c} \mathrm{GEI}2000\\ \ \\ \mathrm{GEO} \end{array} \right.$
Velocity (vel)	
IGRF-14 (igrf)	

Support Data	
Variable	Cadence
Coupling Matrix (matrix)	Once per orbit
Offsets (offset)	Once per orbit
Tweaker (tweaker)	Same as Science Data

The data cadence will be different for the Science Data, the Ephemeris Data, and the Support Data. Science Data will be at 128 sps in the ROI and 16 sps in the BOR. The cadence of the Ephemeris Data is to be determined but can be at a lower rate than the Science Data, given the smoothly varying nature of the ephemeris. The IGRF will initially be calculated at the same cadence as the ephemeris, being interpolated when included as part of the Science Data. Lastly, the Support Data will be at two different cadences. We expect to provide the coupling matrix and offsets at a once per orbit cadence, being interpolated from the calibration files generated during the weekly updates. The tweaker data are currently expected to be at the same cadence as the Science data, although a lower cadence (e.g., two spins) is not out of the question, if file size becomes an issue.

We should note that at the time of writing, the data to be included in the L2 files has not been finalized. Furthermore, once the data are made public, feedback from the scientific community may require the inclusion of other data products.

## Additional Science Opportunities

The primary goal of the TRACERS mission is to investigate the signatures of reconnection at the low altitude dayside cusp. In this section we describe two additional science opportunities. The first is an investigation of one of the consequences of electromagnetic and particle energy input to the ionosphere in the cusp region: ion outflow. The second takes advantage of the high-resolution TRACERS instrumentation to resolve the different types of auroral forms and associated current carriers in the nightside ionosphere. These topics are also addressed by Powers et al. (2025), who describe a student rocket campaign to measure escaping ions above the TRACERS spacecraft altitude, and Dorelli et al. (2025), with their discussion of signatures in the nightside ionosphere that are a consequence of magnetotail reconnection. In describing Science Objective 3 (SO3), Miles et al. (2025a) also note that improving our understanding of ion outflow processes will benefit through the knowledge gained under SO3.

### Ionospheric Ion Ouflows

Yau and André (1997) present an extensive review of the observations of ion upwelling and outflow through ∼30 years of spacecraft observations. One aspect of the observations that warrants emphasizing is that not all observations of upflowing ions necessarily correspond to ions that escape to the magnetosphere. This is especially the case for heavier ions such as oxygen. Indeed, for an escape velocity of 10 km/s, O^+^ ions must generally have an energy of > 10 eV to ultimately escape from the ionosphere. Heavy ions of ionospheric origin initially have energies < 1 eV, and a multi-step process is usually required for these ions to achieve escape energies. This was explored by Strangeway et al. (2000, 2005) using FAST data, and we will summarize some of their results here to emphasize how TRACERS can further our understanding of ion outflows.

**Fig. 14:**
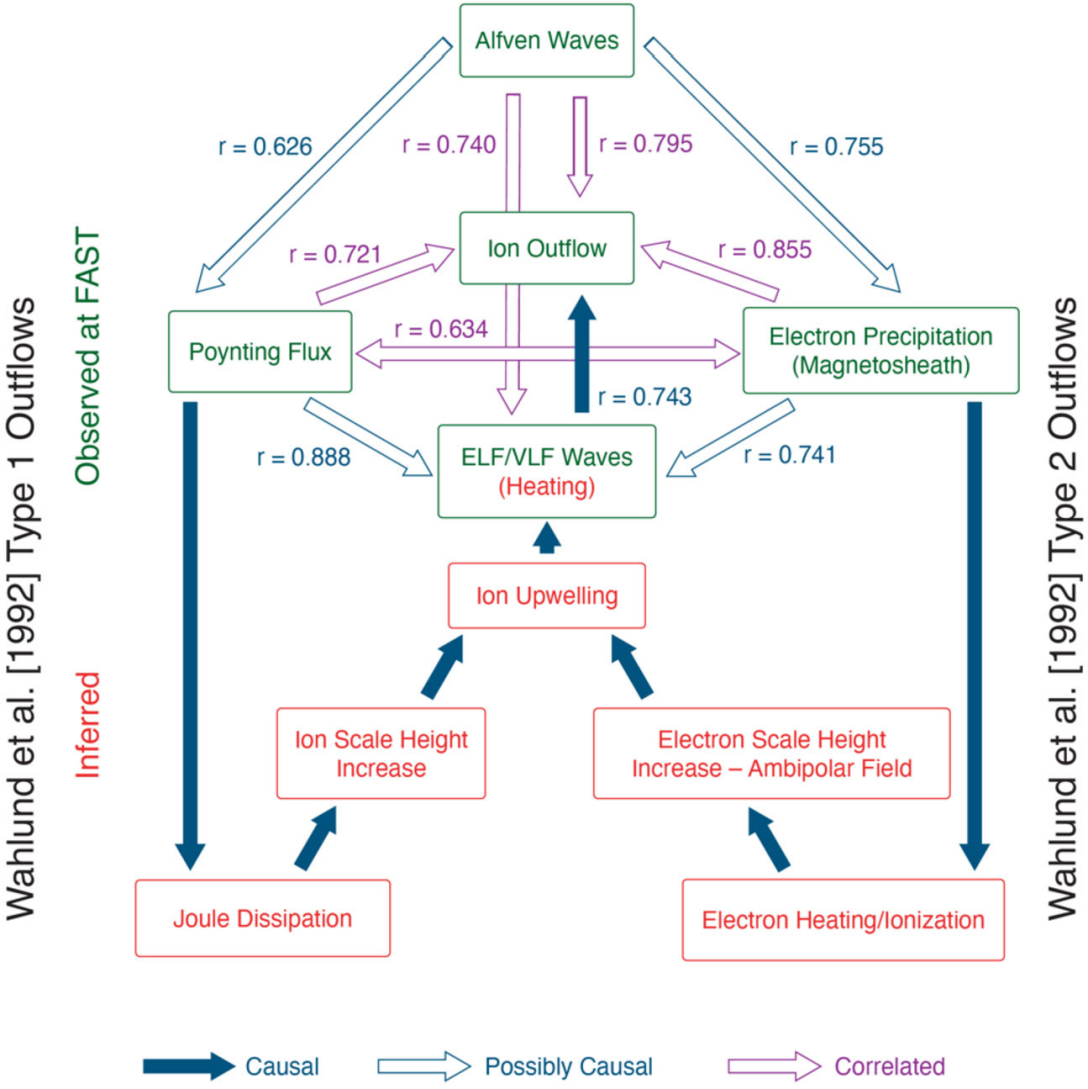
Ion outflow connections

Figure 14 is an updated version of Fig. 1 in Strangeway et al. (2005) that includes Alfvén waves when showing the connections between various phenomena. Strangeway et al. (2005) investigated how low frequency (“DC”) Poynting flux (left-hand side of the figure) and electron precipitation (right-hand side) where correlated with outflows. But the figure also emphasizes that at FAST altitudes the actual connections were by inference.

Considering the left-hand side first. The inferred mechanism is that the Poynting flux deposits energy into the ionosphere through Joule dissipation of the Pedersen currents that act as closure currents for the FACs that carry the downward Poynting flux. In Sect. 2, Fig. 1, we noted that the low frequency (< 0.1 Hz) electric and magnetic fields were related through the height-integrated Pedersen conductivity. At the height where the Pederson conductivity is maximum the ions are highly collisional, while the electrons are essentially collisionless. Additionally, the neutral density is much higher than the plasma density at those altitudes, and the Joule dissipation raises the temperature of the ions (Banks and Kockarts 1973; Strangeway 2012). The resultant scale-height increase results in upwelling of the ions, and this has been referred by Wahlund et al. (1992) as Type 1 outflow in EISCAT radar observations (as noted above, we have chosen to reserve the term outflow to those ions that have achieved escape velocity).

The right-hand side of Fig. 14 corresponds to Type 2 outflows in the parlance of Wahlund et al. (1992). In this case the electron precipitation tends to heat electrons in the F-region, which results in an ambipolar field that again increases the ionospheric scale height. But, as with the left-hand path, the upwelling is insufficient to cause outflows. Consequently, wave heating is invoked. Strangeway et al. (2005) considered ELF waves as the likely source, but this assumption should be revisited.

The box at the top of the figure, labeled “Alfven Waves” has been added to include the pathway investigated in simulations by Brambles et al. (2011). As with all the connections shown the correlation coefficients are comparable. But we have indicated that Alfvén waves could contribute to outflows by either adding to the ionospheric Joule heating, or by enhancing the electron precipitation. Much of the discussion in the student rocket paper (Powers et al. 2025) emphasizes the electron precipitation path, rather than the ionospheric Joule dissipation path, as do Miles et al. (2025a).

The emphasis on Alfvén waves as a source of ion outflows can be thought of as a direct consequence of the work of Wygant et al. (2002) and Chaston et al. (2007), both of which emphasize that Alfvén wave Poynting flux is transferred to precipitating electrons. Chaston et al. (2007) found that at FAST altitudes near the cusp 50% of the energy deposited by electrons was driven by Alfvén wave energy. Chaston et al. (2007) also estimated that 40% of the cusp-region ion outflows were Alfvén wave driven. Hatch et al. (2020) performed a regression analysis, similar to that of Strangeway et al. (2005) and Brambles et al. (2011), with the emphasis on the contribution of Alfvén waves to ion outflows. We do note, however, that Fig. 14 shows two possible drivers of outflows. This is where TRACERS can improve our understanding of the causes of ion upwelling and outflow.

Type 1 outflows are associated with ion heating in the E-region, peaking where the ion-neutral collision frequency matches the corresponding ion gyro-frequency. In Sect. 2 we noted that Alfvén waves with sufficiently long parallel wavelength will be in the near-field region, where the ${E} / {\delta B}$ ratio depends on $\Sigma _{p}$, rather than the Alfvén speed. The distinction between Alfvénic and quasi-static is moot for upwelling associated with Joule dissipation. In addition, the Poynting flux associated with large scale FACs is usually orders of magnitude larger than that carried by low frequency Alfvén waves.

For Type 2 outflows, however, Alfvén wave energization of electrons may be an important contributor to the electron scale-height increase. Stronger correlation between Alfvénic Poynting flux and electron precipitation at TRACERS altitudes relative to that found for FAST would be an indicator that energy transfer from waves to electrons at higher altitudes is important for enhanced outflows.

The topic of ionospheric ion outflow will be revisited in the next sub-section, where we discuss nightside auroral-zone FACs. Scaling laws for nightside outflows have been obtained by Zhao et al. (2022). They used TEAMS data (Klumpar et al. 2001) to derive scaling laws for H^+^ and O^+^ separately. Zhao et al. (2020) also used TEAMS data to re-analyze the dayside cusp data used by Strangeway et al. (2005), extending the scaling laws to include mass composition.

In closing this sub-section, we note that the OCHRE student rocket (Powers et al. 2025) is an ideal complement to TRACERS, and specifically allows real-time comparison of energy fluxes, upwelling, and possible acceleration to escape energies.

### Nightside Auroral Current Structure

Dorelli et al. (2025) discuss an additional science question that TRACERS measurements could be used to study: the low altitude signatures of nightside reconnection. They point out that this would mainly use data acquired in the southern nightside auroral zone because of the tilt of the spacecraft being such that the background magnetic field is closely aligned to the spin axis in the northern dayside cusp. From a MAG perspective this would also present an opportunity to investigate different types of FAC found in the nightside ionosphere. As with the previous sub-section, we will use FAST data to show examples of different FACs.

**Fig. 15:**
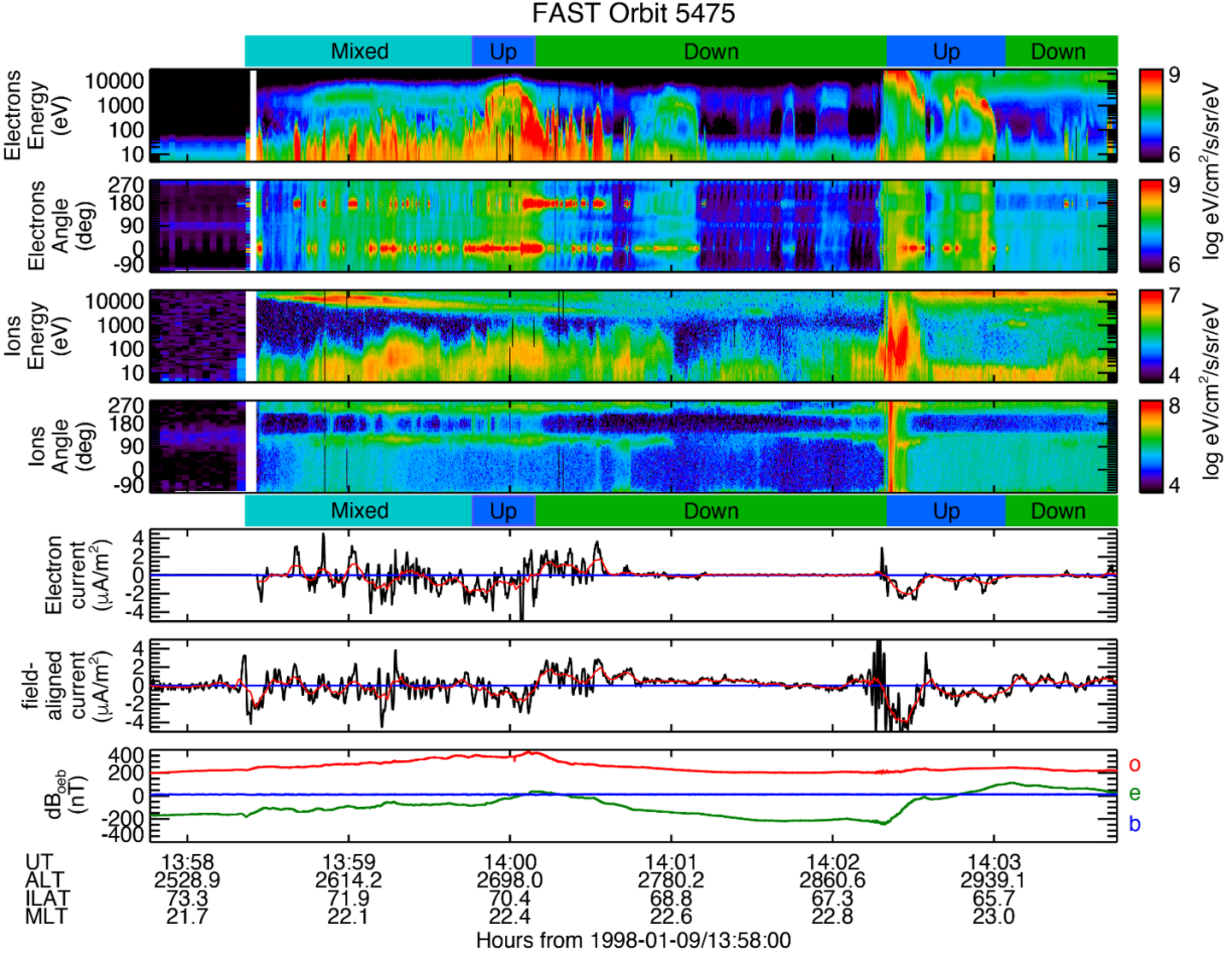
Nightside auroral zone data from FAST

Figure 15 shows data acquired by FAST around 14:00 UT on January 9th, 1998. We note that a modified grey-scale version of this figure was published as Fig. 11.7 in Russell et al. (2016). From top to bottom the figure shows electron Differential Energy Flux (DEF) as a function of energy; electron DEF as a function of pitch angle; ion DEF as a function of energy; ion DEF as a function of angle; field-aligned current derived from the net electron number flux; field-aligned current from the magnetometer data, assuming a infinite sheet current; and the magnetic field perturbation in Outward East B-field-aligned coordinates (oeb). At high latitudes in the northern hemisphere the Outward direction points to the northern magnetic pole.

In addition to the data themselves, we have indicated three different types of FACs by the blue, green, and cyan boxes labelled Up, Down, and Mixed, respectively. The different types of FAC were identified based on the running average of the magnetic-field derived FAC, shown in red. Note that a downward FAC is positive because the ambient magnetic field points down in the northern hemisphere auroral zone. Upward FACs are mainly carried by precipitating electrons, with downward FACs carried by upward-going electrons.

In the two pitch angles plots we use the angular range from -90° to +270°. This ensures that upgoing and downgoing loss cone are clear. Looking at the ions, for example, ion conics are observed most of the time. An ion with a pitch angle of 180° is flowing up the field line, away from the ionosphere, and there are clear maxima in the ion fluxes around 180° ± 40°, outside of the loss cone, corresponding to an ion conic. The angular width of the loss cone width changes. As we note below, the loss-cone tends to be wider when electrons are accelerated out of the ionosphere. We note that the loss cone centered on 0° is filled. This is presumably because of pitch-angle scattering at higher altitudes. The upgoing ions have energies above 10 eV, and while we have not determined if the ions are H^+^ or O^+^, they can escape. The sudden increase in energetic ion fluxes after the data gap at the left of the figure is where FAST transitions from lobe (open) to plasma sheet boundary layer (closed) field lines. Being on closed field-lines the escaping ions will contribute to the plasma sheet plasma.

Turning now to the electron observations. The electrons above 1 keV with DEF around 10^7^ $\mathrm{ev}\ (\mathrm{cm}^{2}\, \mathrm{s}\, \mathrm{sr}\, \mathrm{eV})^{-1}$ (green in the spectrogram) are plasma sheet electrons. Notably, where we observe more intense fluxes above a keV is where we observe upward-directed FACs. Just after the start of the second interval of upward current the DEF as a function of pitch angle is relatively uniform outside of the loss-cone. If we plotted the data using a contour plot as a function of pitch angle we would see what is known as a horseshoe distribution. This is known to be a source of Auroral Kilometric Radiation (AKR) (e.g., Strangeway et al. 2001). But elsewhere in the regions of upward current there appears to be a relatively narrow beam near 0°. This suggests an additional source of electrons within a temperature of the order 100 eV or less, besides the relatively high keV energy plasma sheet electrons.

The narrow pitch angle electrons are also observed in the first interval of upward current. But, at times, there are upgoing and downgoing electrons occupying a relatively narrow pitch angle range. Narrow pitch angle upgoing electrons are observed at the start of the first region of downward current. But for most of downward current the currents are relatively weak, and maybe being carried by electrons below the 6 eV instrument cut-off (see Carlson et al. 2001 for a description of the FAST electrostatic analyzers). To make this clear, upward accelerated electrons of ionospheric origin are the likely downward current carriers. If we assume an electron density of 10 cm^−3^, and bulk streaming energy of 1 eV, then these electrons carry 1 $\mu $Am^−2^ of current, more than the current density after 04:00:50 UT and before 04:02:00 UT as shown in the second panel from the bottom of Fig. 15.

The more intense upward electrons have energies up to a few 100 eV. It is not clear how they became so energized. It is possible that they were accelerated by quasi-static electric fields, but some form of wave activity must be involved to spread the fluxes in energy. This is one area that TRACERS data will be particularly useful, as TRACERS will be at an altitude where the initial acceleration is taking place.

Understanding the processes that occur within the region of mixed current will also benefit from TRACERS observations. This region is often observed at the higher latitudes corresponding to the boundary layer plasma sheet, but need not be restricted to these latitudes. The dominant feature in the electron spectra is the presence of narrow pitch angle electron distributions that are within the loss-cone of both upgoing and downgoing particles. TRACERS can provide insight into how the electrons are accelerated out of the ionosphere. With TRACERS passing through the auroral zone so frequently it would be worth exploring opportunities for coordinated studies with higher orbiting spacecraft to address how the upflowing electron distributions evolve, and what is the source of the downgoing electrons. One highly speculative idea is that we are observing the signature of interhemispherical FACs, possibly from bouncing Alfvén waves, analogous to the Alfvén wings observed at Jupiter (Bagenal 1983) and simulated by Su et al. (2006). For the terrestrial case it may be that enhanced reconnection-driven flow channels come into equilibrium with the ionosphere through bouncing Alfvén waves.

In closing this section, we note that Chaston et al. (2008) call the region of “Mixed” currents “Alfvénic”. They further argue that the waves are turbulent, and have short perpendicular wavelength, such that the observed frequency of the waves is a manifestation of Doppler shift by the spacecraft motion through the plasma. Indeed, if the perpendicular wavelength becomes comparable to the plasma skin-depth (i.e., ${k_{\bot} c} / {\omega _{pe}} \gtrsim \mathrm{O}$(1), where the symbols have their usual meaning), then the waves may carry a parallel electric field. This arises out of non-ideal-MHD terms in the electron fluid momentum equation. These Alfvén waves are often referred to as “inertial”. Returning to our earlier discussion of ion outflows, electron acceleration by the parallel electric field of inertial Alfvén waves will contribute to Type 2 outflows. Again, TRACERS observations of the nightside auroral zone will further our understanding of the different FAC regions shown in Fig. 15, and the types of ionospheric outflows found there.

## Concluding Remarks

In this manuscript we have described the TRACERS fluxgate magnetometers (MAG) in detail, including the heritage, design and implementation for TRACERS, pre-launch testing, and the approach to calibration using pre-launch tests for preliminary calibration and post-launch on-orbit calibration. The post launch calibration is also part of the process by which low-level (L1) data are converted to science-grade L2 data that are available for public use.

The TRACERS MAG can contribute to all the science objectives of TRACERS, but with specific application to measurement of the magnetic field associated with both large-scale (quasi-static), and smaller scale (Alfvénic) Field-Aligned Currents (FACs).

We have also discussed additional science opportunities beyond the three primary science objectives, specifically addressing the observation that ions of ionospheric origin can be accelerated to escape energies, and the closely related topic of the types of FACs that flow into and out of the ionosphere. The latter also has bearing on outflows, as the electromagnetic and particle energy flowing into the ionosphere can follow multiple pathways. The TRACERS instrument suite is of such high quality that it can make a significant contribution to our understanding of FACs in the nightside auroral zone, as well the dayside cusp. Indeed, comparing and contrasting observations from dayside and nightside may provide a major insight into the underlying physical processes that result in particle precipitation and ionospheric escape, given the evidence that large-scale and Alfvénic FACs can occur at different locations in the nightside auroral zone, while they tend to overlap in the dayside cusp.
